# Modeling Performance of Microservices Systems with Growth Theory

**DOI:** 10.1007/s10664-021-10088-0

**Published:** 2022-01-11

**Authors:** Matteo Camilli, Barbara Russo

**Affiliations:** Faculty of Computer Science, Free University of Bozen-Bolzano, Bolzano, Italy

**Keywords:** Performance requirement, Time series analysis, Point processes, Growth theory, Microservices systems

## Abstract

**Context:**

The microservices architectural style is gaining momentum in the IT industry. This style does not guarantee that a target system can continuously meet acceptable performance levels. The ability to study the violations of performance requirements and eventually predict them would help practitioners to tune techniques like dynamic load balancing or horizontal scaling to achieve the resilience property.

**Objective:**

The goal of this work is to study the violations of performance requirements of microservices through time series analysis and provide practical instruments that can detect resilient and non-resilient microservices and possibly predict their performance behavior.

**Method:**

We introduce a new method based on *growth theory* to model the occurrences of violations of performance requirements as a stochastic process. We applied our method to an in-vitro e-commerce benchmark and an in-production real-world telecommunication system. We interpreted the resulting growth models to characterize the microservices in terms of their transient performance behavior.

**Results:**

Our empirical evaluation shows that, in most of the cases, the non-linear S-shaped growth models capture the occurrences of performance violations of resilient microservices with high accuracy. The bounded nature associated with this models tell that the performance degradation is limited and thus the microservice is able to come back to an acceptable performance level even under changes in the nominal number of concurrent users. We also detect cases where linear models represent a better description. These microservices are not resilient and exhibit constant growth and unbounded performance violations over time. The application of our methodology to a real in-production system identified additional resilience profiles that were not observed in the in-vitro experiments. These profiles show the ability of services to react differently to the same solicitation. We found that when a service is resilient it can either decrease the rate of the violations occurrences in a continuous manner or with repeated attempts (periodical or not).

**Conclusions:**

We showed that growth theory can be successfully applied to study the occurences of performance violations of in-vitro and in-production real-world systems. Furthermore, the cost of our model calibration heuristics, based on the mathematical expression of the selected non-linear growth models, is limited. We discussed how the resulting models can shed some light on the trend of performance violations and help engineers to spot problematic microservice operations that exhibit performance issues. Thus, meaningful insights from the application of growth theory have been derived to characterize the behavior of (non) resilient microservices operations.

## Introduction

Over the last few years, microservices emerged as the architectural style of choice in a variety of application domains in the IT industry. Loosely, the microservices paradigm is an approach for developing a single application as a suite of small black-box services, each running in its own process and adopting lightweight messaging protocols like HTTP/REST (Lewis and Fowler 2014). These services are built around business capabilities and they are independently deployable by automated procedures inside “continuous development/delivery” pipelines. As stated by Taylor et al. (2009), in this context, available architecture alternatives and their parameters lead to a non-trivial space of architecture deployment configurations to choose from. Recent studies confirm that these choices significantly influence the performance and scalability of microservices systems. Moreover, improper deployment environment settings and misconfigurations are often root causes of transient performance degradation that are difficult to predict especially under unforeseen circumstances like workload spikes (Ueda et al. 2016; Avritzer et al. 2020b). As stated by Soldani et al. (2018), there exists a growing scientific interest in the microservices architectural style, in particular, for what concerns performance modeling, analysis, and prediction. Recent research focuses on the detection of performance violations and the study of their occurrences over time. A *performance violation* is a condition occurring whenever a given performance requirement is (temporary) not satisfied. A performance requirement is typically defined according to usability engineering practices (Nielsen 1994) or explicit contracts with the customer (e.g., service level agreement). Nevertheless, issues arise when the performance requirement or the information needed to determine it is unavailable or not accessible. According to Jiang and Hassan (2015), this is a common scenario. Thus, we propose to adopt the method introduced by (Avritzer et al. 2018) to automatically derive the performance requirement by empirically estimating the threshold value which defines the acceptable behavior of each microservice.

The major goal of this work is to provide an novel instrument that allows analyze the performance violations of microservice operations over time, describe their resilience and eventually spot operations that may not be able to recover from a degraded operating mode.

We introduce a novel approach to model the occurrences of performance violations of microservices systems based on stochastic models of *growth theory*, which have been for long successfully used in the context of reliability analysis (e.g., Virene (1968), Abdel-Ghaly et al. (1986), Musa et al. (1987), Succi et al. (2003a), Taber and Port (2014), Rossi et al. (2010), Taber and Port (2016), and Kumar et al. (2019)) and more recently to describe the COVID-19 pandemic (Shen 2020). In our work, we use such a theory to model the occurrences of *performance violation* s in service operations over time. To the best of our knowledge, this is the first time growth theory of point processes is exploited to model performance violations. Specifically, our approach models the occurrences of performance violations of each microservice operation of a System Under Test (SUT) as a point process (or counting process) that describes the cumulative number of violations over time.

To apply our approach, we conducted an empirical evaluation by carrying out both in-vitro and in-production controlled experiments. In-vitro experiments were conducted by testing a microservice system benchmark called Sock Shop with synthetically generated end-users. In-production experiments were conducted by monitoring a real-world Telecommunication service-based system developed by Ericsson and deployed in their production environment while interacting with real end-users. In both cases, we first defined a quantitative threshold expressing the *performance requirement* (Avritzer et al. 2020a), and then we collected violations of this threshold for each microservice operation over time. Then, we fit nine state-of-the-art non-linear growth models on the datasets of microservice operations and then we compared them in terms of four estimation accuracy and prediction ability. The results of our empirical evaluation show that growth models can be derived and calibrated with limited effort. We found that the average execution time per individual calibration process is low and it varies from less than a second to about 1 minute. We also found that, for most of the microservices, finite non-linear growth models can describe the occurrence of performance violations better than linear models when the target service operation is resilient, i.e., the operation eventually restores acceptable performance levels even under changes of the nominal workload conditions. Considering both estimation and prediction ability, we also observed that S-shaped growth models are the most accurate for a large number of operations, but not for all. For some operations, we have indeed found that linear models are better. The operations whose violations follow a linear model do not exhibit a resilient behavior. Thus, they require attention from engineers.

The major contributions of this work can be summarized as follows:

1. novel *modeling approach* for the analysis of transient performance behavior of microservices, grounded on stochastic models of growth theory applied to collect, model, analyze, and interpret performance violations over time;
2. *holistic methodology* that drives the mechanical creation of multiple growth models and then the selection of the most appropriate model(s) able to estimate and predict performance violations over time with the highest accuracy;
3. *empirical evaluation* through controlled experiments using in-vitro and in-production environments that aim at studying the cost-effectiveness of the model calibration process, the accuracy of alternative growth models, the insights that can be extracted from the models, and the applicability as well as the generalization of the findings to real-world systems running in production.

The remainder of the paper is structured as follows. In Section 2 we introduce an overview of our methodology. In Section 3 we introduce our research questions and the preliminary stages of our methodology (experiment design and execution). In Section 4, we detail the core stage of our methodology used to derive the growth models that describe the performance violations (model learning and selection). In Section 5, we describe the major experimental results obtained from the execution of controlled experiments and answer our research questions. In Section 6, we discuss threats to validity. In Section 7, we discuss related work. Finally, in Section 8, we report our conclusion and future directions of this work.

## Methodology

In this section, we introduce the main stages of our methodology that guide the generation of the growth models. We start defining the performance requirement that allows us to compute the violations (Section 2.1). Then, we illustrate the two core stages of the methodology: *experiment design and execution* (Section 2.2); and *model learning and selection* (Section 2.3). We conclude this section by discussing the prerequisites we assume on a target system that allows our methodology to be replicated (Section 2.4).

### Performance requirement and violation

The notion of *performance requirement* refers to the capability of the target microservices system to handle requests within time constraints. We measure the performance of an operation by its response time. Its performance requirement is defined by its response time being smaller than a given *performance threshold*. To compute the threshold, we leverage the domain-based metric approach introduced in Avritzer et al. (2018). We first determine a *reference* workload (or load) and deployment architecture 〈*λ*_0_,*α*_0_〉. We assume that the system deployed with enough resources and accessed by a minimal number of concurrent users is responsive, that is the response time of individual operations is acceptable. We then evaluate the SUT under a *target* load and deployment architecture 〈*λ*,*α*〉 and compare the performance under the reference and target settings. To this aim, we first perform a load test session under the reference setting 〈*λ*_0_,*α*_0_〉 to compute the average response time *μ*_*j*_(*λ*_0_,*α*_0_) and standard deviation *σ*_*j*_(*λ*_0_,*α*_0_) of the requests to each operation *o*_*j*_. Thus, we define the *j*^*th*^ *performance threshold*, as follows:

1 $$  {\Gamma}_{j}(\lambda_{0},\alpha_{0})=\mu_{j}(\lambda_{0},\alpha_{0}) +3 \cdot \sigma_{j}(\lambda_{0},\alpha_{0}).  $$

Then, we perform a new load test session under the target setting 〈*λ*,*α*〉. We say that that the operation *o*_*j*_ *violates* the performance requirement at time $\bar {t}$ if:

2 $$  \mathit{RT}_{j}(\bar{t}) > {\Gamma}_{j}(\lambda_{0},\alpha_{0})   $$

with $\mathit {RT}_{j}(\bar {t})$ the response time of operation *o*_*j*_ at $\bar {t}$. Equation (1) and (2) can be explained by the Chebyshev’s inequality (Pukelsheim 1994; Ibe 2013), which represents a non-parametric version of the well-known 3 ⋅ *σ* empirical rule. The Chebyshev’s inequality states that at least 88.8*%* of the values in a univariate distribution lie within three standard deviations from the mean. Thus, a response time above the threshold Γ_*j*_, as in (1), represents an outlier behavior detected as a *performance violation*. This approach falls in the category of strategies referred to as *response time percentiles* as discussed by Wert (2018). Such a strategy allows a substantial amount of violations to be detected in a short time and therefore, the non-linear regression analysis to be carried out with statistical significance even after a small observation period.

### Experiment design and execution

**Fig. 1:**
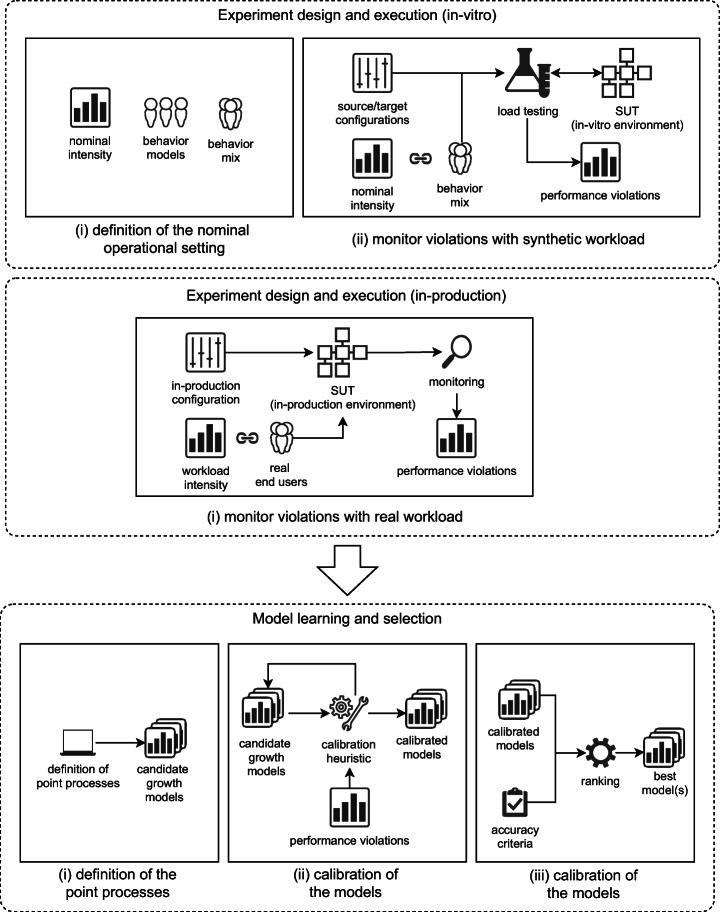
Overview of the main stages of our methodology

According to the schema in Figure 1, the first stage can be conducted using either an in-vitro or in-production setting. As detailed in the next sections, to evaluate our methodology we considered both in-vitro and in-production settings. We first executed a set of in-vitro controlled experiments on a demo system and then we replicated the experiments with a real-world in-production system. The in-vitro experiments allow us to control variables and repeat observations, while the in-production experiments allow us to perform our methodology in a real-world environment and show its applicability in industrial contexts.

#### In-vitro experiments

In this case, we follow two steps to define the expected nominal operational setting and then execute the proper load testing sessions that feed the second stage *model learning and selection*.

1. *Definition of the nominal operational setting.* This step defines the execution context that is likely to happen in production. According to Avritzer et al. (2020a), this context is defined through a *workload specification model* from which user behavior models are derived and then mixed according to frequencies as defined by the so-called *behavior mix*. A *workload intensity* (i.e., number of concurrent users) is then selected to represent the nominal workload that is expected in production.
2. *Monitor violation with synthetic workload.* This step reproduces a *demanding* operational setting to stress the microservices and then analyze their performance. The demanding setting is created by augmenting the nominal one with changes in the number of concurrent users. Such changes are meant to add noise and emulate a production setting in which the number of concurrent users is not fixed and may increase unexpectedly. Two *load testing* sessions in our in-vitro environment are then performed to collect the data to fit the performance models. In particular, we collect the *performance violations* based on the definition of *performance requirements* of the SUT. The outcome of this step is the log of all the performance violations per individual operation invoked for all microservices.

#### In-production experiments

In this case, the methodology follows a single step to monitor the target system in production while interacting with real users. The monitoring activity feeds directly the second stage *model learning and selection*.

1. *Monitor violations with real workload.* Rather than defining and reproducing a synthetic operational setting using an in-vitro environment, we monitor the target system in-production while interacting with real end-users that stress the microservices under a demanding operational setting. In this case, the reference and target deployment architectures are the same. As we could not know the number of concurrent users, we monitor the microservices under different rates of server utilization and we collect response time data in order to detect occurring performance violations over time.

### Model learning and selection

The second stage takes as input the outcome of the experiment execution and it analyzes the performance violations to build the candidate growth models and then select the best one(s). This process follows three main steps reported below.

1. *Definition of the point processes.*
  After collecting runtime evidence from the first stage, we identify the candidate *point processes* that model the performance violations of the target microservices over time. Here, we adopt growth theory (Rigdon and Basu 2000) to model events (i.e., performance violations) over time as stochastic processes, in order to analyze and predict future scenarios. The outcome of this stage is a set of candidate models that represent different possible performance evolution scenarios.
2. *Calibration of the performance models.* In this phase, we tune the selected models by fitting them on the available data in order to determine the model parameters that best describe the empirical evidence. As stated by Virene (1968), the initial choice for model parameters is not trivial. This choice often determines the ability to converge in the fitting process. Thus, we propose an automated strategy to identify the initial values of the parameters and control the regression procedure of the candidate models.
3. *Selection of the top performance models.* This last step aims at evaluating the models through a systematic comparison. Namely, we evaluate the calibrated models based on the ability to fit the experimental data by means of four state-of-the-art metrics of *accuracy* (i.e., the ability to model the occurrences of performance violations) and *prediction ability* (i.e. the ability to predict future violations). The outcome of the comparison produces the best performance models for each individual microservice or microservice operation of the target system from which engineers can extract insights and identify resilience issues.

### Prerequisites

Our approach can be applied to any service-based system provided that some prerequisites are satisfied. Such prerequisites allow the response time to be collected and the performance behavior to be monitored for each service or service operation of interest. This ensures that any performance issues can be localized at service or lower level. In the following, we list the prerequisites that shall hold to apply our methodology. It is worth noting that microservices systems meet all of them. In general, the approach can be also applied to systems having a Service Oriented Architecture (SOA) as long as the below prerequisites hold. In the in-vitro environment, we were able to perform a study at the level of microservice operations, whereas in the in-production environment we performed the study at the level of individual microservices.

- a target SUT is a service-based system in which each service exposes one or more operations accessible through a RESTful API or SOA Protocol;
- each service implements a single business capability within a bounded context (Richardson 2018);
- the SUT is deployed onto an in-vitro or in-production environment supporting the execution of services upon one or more virtual/bare-metal machines;
- the response time of individual client requests can be measured and recorded at the lowest available granularity level (either service-level or operation-level);
- measurable solicitation can be applied to the target systems (workload intensity, server utilization, or alike) in two different operational settings: a reference (ideal) setting used to extract performance requirements, and a demanding setting used to monitor the performance violations.

## Experiment design and execution

In this section, we first introduce the research questions (Section 3.1). Then, we present the design and execution of the experiments we conducted using in-vitro and in-production settings (Sections 3.2 and 3.3, respectively) to answer our research questions.

### Research questions

The major goal of this work is to study growth theory in the context of performance modeling of microservices systems and the detection of resilience issues. To this end, we aim at answering the following research questions.

**RQ1:** *What is the cost-effectiveness of the proposed calibration heuristic for fitting growth models?* We study both the cost, in terms of execution time, and the effectiveness, in terms of success ratio of the calibration process, considering all the state-of-the-art growth models in Table 10 describing the occurrences of performance violations.

**RQ2:** *To what extent do the proposed non-linear growth models accurately estimate the occurrences of performance violations of microservice operations?* We study to what extent state-of-the-art growth models can represent the occurrences of violations of performance requirements.

**RQ3:** *What are the insights we can derive from growth theory applied to the study of performance violations of a microservice system?* We study whether there exists a model that, more than others, accurately represents the occurrences of performance violations. In such a case, the model characteristics may help interpret the performance of the operations over time and eventually elevate attention on microservices that yield severe performance issues.

**RQ4:** *To what extent our methodology can be applied to real-world in-production systems?* We study the applicability of our methodology in a real-world system interacting with end-users in production to further extend and generalize our findings. The production environment of real-world systems typically add challenges such as the lack of control over factors of interest and settings of the SUT.

### In-vitro everxperiemnt

#### System under test

We adopt a microservices system called Sock Shop^1^. Sock Shop is considered a microservice reference application used by researchers in the field of performance engineering to evaluate their approaches, e.g., see the studies by Benni et al. (2020), Assunção et al. (2020), and Grambow et al. (2020), to name a few. The system implements a containerized e-commerce web application composed of 19 microservice operations (Table 1) implemented by using heterogeneous technologies (e.g., Java, .NET, Node.js, and Go). The services expose REST APIs listed in Table 1.

**Table 1:** Operations exposed by microservices of Sock Shop

Label	Path (relative)	Method
addToCart	/cart	POST
basket	/basket.htm	GET
catalogue	/category.html	GET
cataloguePage	/catalogue?page = {}&size = {}	GET
catalogueSize	/catalogue/size?size= {}	GET
createOrder	/orders	POST
getAddress	/address	GET
getCard	/card	GET
getCart	/cart	GET
getCatalogue	/catalogue	GET
getCustomer	/customers/{}	GET
getItem	/catalogue/{}	GET
getOrders	/orders	GET
getRelated	/catalogue?sort = {}&size = {}&tags = {}	GET
home	/index.html	GET
login	/login	GET
showDetails	/detail.html?id = {}	GET
tags	/tags	GET
viewOrdersPage	/customer-orders.html	GET

The users interact with the application through a web user interface. As an example, a *buyer* is likely to follow these steps: visit the home page, execute the authentication, view the catalog and some details, add one or more product to the cart, and then create an order. This usage pattern reduces to a path of service invocations. For instance, surfing the catalog and adding products to the cart can be executed through the following path of valid requests: , , , , , and . Instead, a nominal *visitor* is likely to surf the catalog without authenticating and filling up the cart. In general, different user behaviors yield various request invocations that mixed with a particular workload intensity compose the workload of the SUT.

#### Behavior specification

**Fig. 2:**
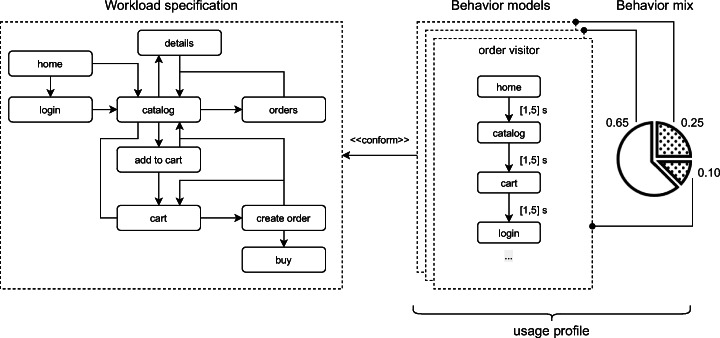
Example of usage profile defined through the workload specification model, a number of behavior models, and the behavior mix

The approach used to define the behavior of the synthetic users in terms of session-based interactions with the SUT is adopted by Avritzer et al. (2020a), Avritzer et al. (2018), and Vögele et al. (2018). In particular, the specification of the behavior consists of a number of elements that are detailed below and illustrated in Figure 2:

- a *workload specification model*, in terms of the allowed sequences of requests to microservice operations;
- a set of *behavior models*, representing user sessions in terms of valid operations and a pseudo-random think time between subsequent invocations defined according to the aforementioned workload specification model;
- a *behavior mix*, in terms of probabilities (frequencies) associated with each behavior model to occur during workload generation.

#### Automated load testing

This process generates load tests and executes them on the SUT with synthetic users according to a given operational setting in an automatic manner. Each user *u*, described by a behavior model, operates during a test session with probability weight *ω* defined by the behavior mix. The set of synthetic users and their weight values define the *usage profile*, as shown in Figure 2. The usage profile is formally defined as follows:

3 $$  {\Omega} = \{ \langle \omega_{1}, u_{1} \rangle, \dotsc, \langle \omega_{h}, u_{h} \rangle \}.   $$

where *u*_*i*_ is a behavior model that conforms to the workload specification model, and *ω*_*i*_ is the corresponding weight as defined by the behavior mix. The workload is then generated by a weighted random sampling of synthetic users that behave according to the models. Load tests are executed under a given workload intensity in terms of number of concurrent users. The output of a test session is a triplet (*μ*_*j*_,*sd*_*j*_,*ν*_*j*_) consisting of 1) the mean of the response time, the standard deviation of the response time, and the invocation frequency of each operation *o*_*j*_.

Automation has been implemented using the PPTAM testing framework^2^ introduced by Avritzer et al. (2019) and Avritzer et al. (2021). PPTAM handles the generation and execution of the tests and it automates the deployment/undeployment of the SUT. This orchestration layer relies on a user-friendly DSL that specifies the test sessions in a declarative manner through a number of parameters:

- usage profile Ω (behavior models and behavior mix);
- selected workload intensity *λ*;
- deployment configuration *α* including the amount of RAM, CPU share, and replicas per microservice.

**Fig. 3:**
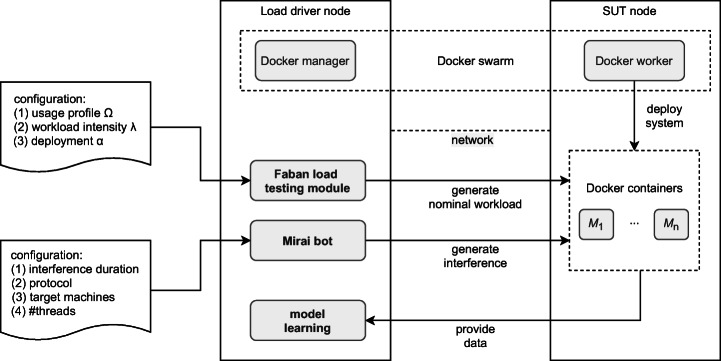
Infrastructure used to automate the in-vitro experiments

Figure 3 shows a high-level schema of the infrastructure used to implement our in-vitro environment for the controlled experiments. As shown by the schema, Docker automates the deployment of the SUT by using the selected configuration *α*. Then, the Faban module is used to generate the nominal workload intensity *λ* according to the usage profile Ω. To augment the nominal workload, we generated pseudo-random changes in the number of concurrent users (i.e., the demanding setting) by adopting the academic version of the Mirai bot (Barker 2016; Antonakakis et al. 2017), which is a modified version of the malware described by Antonakakis et al. (2017). We exploited the capability of the bot to send random HTTP requests to the services exposed by the SUT. As anticipated in Section 2, the additional load is meant to add noise and emulate a realistic environment where the workload can change in an unforeseen way. During the test session, PPTAM logs the response time for each executed request and it feeds the model learning module that builds in turn the candidate growth models.

The two nodes in Figure 3 are containerized virtual machines on top of the VMware ESXi^3^ hypervisor. Two virtual machines have been used for containerized deployment: the *Load driver node* with 4 GB RAM, 1 CPU core at 2600MHz; and the *SUT node*, with 8 GB RAM, 4 cores at 2600 MHz with SSDs. Both machines use an EMC VNC 5400 series network attached storage solution^4^ and are connected using a shared 10 Gbit/s network infrastructure. We rely on Docker CE v17.12 for the deployment of the containerized application on both infrastructures. The Mirai requests are conducted from the load driver server machine, which is configured with 4 GB RAM, 1 core at 2600MHz, and is connected to the SUT server machine through a 1000 MBit network.

#### Test deployment and execution

We executed two test sessions, and . In , we chose and deployed the SUT under the reference setting 〈*α*_0_,*λ*_0_〉. Specifically, we chose:

$$ \begin{array}{@{}rcl@{}}\begin{array}{ll} \alpha_{0} &= \{4 \text{ GB RAM}, 100\% \text{ CPU share}, 1 \text{ replicas for all operations}\}\\ \lambda_{0} &= 2 \end{array}\end{array} $$

assuming that with the highest possible resources and only two users the SUT is responsive at its best. As described in Section 4, the reference setting is used to extract the performance requirements that allow us to record the violations during the second session.

Thus, we performed the second test for which we deployed the SUT for a target setting 〈*α*,*λ*〉:

$$ \begin{array}{@{}rcl@{}}\begin{array}{ll}\alpha =& \{2 \text{ GB RAM}, 25\% \text{ CPU share}, 2 \text{ replicas for } \mathtt{getCart}\}\\ \lambda =& 100 \end{array}\end{array} $$

We halved the RAM and reduced the shared CPU while replicating the container for the most solicited under 100 concurrent users. Such a setting represents an arbitrary target configuration that engineers might want to evaluate under demanding circumstances.

In both Test1 and Test2, we adopted three behavior models (Visitor, Buyer, Order visitor) and the following behavior mix (0.4, 0.3, 0.3), as follows:

- Visitor (*ω* = 0.4): visits the home page, views the catalog and the details of some products.
- Buyer (*ω* = 0.3): visits the home page, logs in, views the catalog and some details, adds a product to the cart, visits the cart, and creates an order.
- Order visitor (*ω* = 0.3): visits the home page, logs in, and views the stored orders.

A *think time* (i.e., the time between the completion of one request and the start of the next one) modeled as exponential distribution (with average inter-arrival time between 1 and 5 seconds) is added to represent a more realistic user behavior, as stated by Avritzer et al. (2020a). The test sessions have been executed by following these steps:

- deployment of the SUT and PPTAM;
- 60-seconds test execution (ramp-up) to reach a steady state;
- 30-minutes test execution under the given load and usage profile;
- collection of response time data for each operation over the 30 minutes session.

**Fig. 4:**
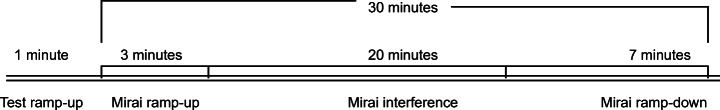
timeline

During , the Mirai bot is launched as a dedicated parallel process after the test rump-up stage, Figure 4. After the warm-up of Mirai (three minutes), the response time is collected until the end of the ramp-down, in which the SUT continues to receive HTTP requests for seven more minutes. The initial ramp-up and the final ramp-down are needed to avoid the interference of other initialization or de-initialization processes eventually running during the execution of the Mirai bot. The Mirai bot is configured with the following parameters:

- 20 minutes (1200 seconds) duration of interference;
- HTTP protocol;
- target IP address of the SUT (i.e., the machine with Sock Shop installed); and
- 256 concurrent threads.

### In-production experiments

#### Deployed system

**Fig. 5:**
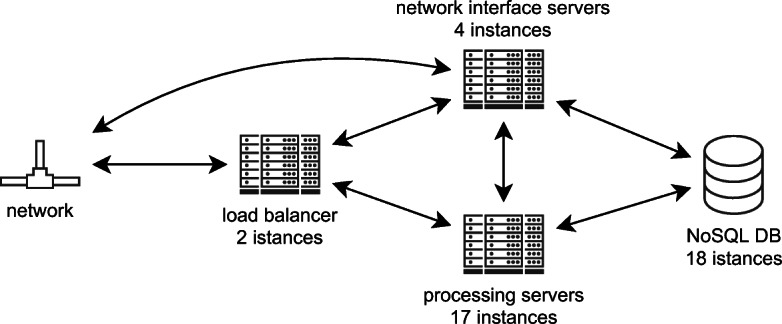
In-production infrastructure of the Telecommunication System

Our target system of choice in this setting is a real-world large-scale telecommunication system developed by Ericsson. The production environment of the company is a cluster of bare metal machines 5. The system is composed of more than 20 subsystems developed by distributed teams using agile practices. The scale of operation makes the system particularly interesting for our investigation (millions of concurrent users per second). Furthermore, it represents a relevant and representative example of a performance-critical service-based system satisfying the pre-requisites listed in Section 2. For this case study, we used a subset of the performance-critical services running onto the processing servers of the infrastructure shown in Figure 5. The system receives requests from the network, which are sent to a load balancer and then forwarded through an API gateway to our subsystems of interest composed of the following main microservices^6^

#### Operational setting and execution

We carried out two experiments to set up the threshold for performance requirements and collect violations. Being an in-production setting, we could not control the workload using synthetic users and define behavior specifications as in Section 3.2.2. We instead monitored the workload of real end-users by measuring the server’s utilization and collect the response time with the monitoring tools provided by the company. For the same reasons, the reference and target deployment architectures for the experiments is the same.

We executed a first session under 40*%* server utilization rate (6.4*k* transactions per second). According to Ericsson’s stakeholders, in this setting the system is assumed to achieve the desired performance level. Thus, we monitored the system and collected the response time to extract the performance requirements for all services following our methodology. In a second session, the response time per individual service was collected by observing the system for $\sim 21$ hours under the utilization rates 50*%*, 60*%*, 70*%*, 80*%*, 90*%*, and 100*%* (up to 16.0*k* transactions per second) to collect the performance violations.

## Model learning and selection

In this section, we describe in detail the second stage of our methodology outlined in Section 2. We describe the point processes used to model the occurrence of performance violations (Section 4.1). Then, we discuss the calibration of these models (Section 4.2). Finally, we present the estimation and prediction measures to compare and rank the models (Section 4.3).

### Point processes of performance violations

As anticipated in Section 2, our approach exploits growth theory traditionally used in software reliability (Virene 1968; Rigdon and Basu 2000; Lyu 1996; Rossi et al. 2010; Port and Taber 2018). According to such theory, occurrences of events are modeled as a stochastic point process. In this work, we design models of performance evolution as stochastic processes where events are the occurrences of performance violations observed during load testing. According to growth theory (Rigdon and Basu 2000), for each operation *o*_*j*_, we can model the occurrences of violations by the random variable *N*_*j*_(*t*) that counts such events as follows:

4 $$  N_{j}(t)=N_{j}((0,t])=|{x\in (0,t]: RT_{j}(x)\geq {\Gamma}_{j}}|  $$

The variable *N*_*j*_(*t*) defines a *counting process*, whose expected mean *E*[*N*(*t*)] is a right-continuous function of global time defined as:

5 $$  f_{j} (t)=E[N_{j}(t)]={\sum}_{n\geq 1} n \cdot P\{N_{j}(t)=n\}  $$

The function *f*_*j*_(*t*) models the expected cumulative number of performance violations of an operation *o*_*j*_ over time. In literature, various analytic expressions for the functions *f* have been proposed. In this work, we consider a number of candidate models traditionally used by the research community to study phenomena of interest in software and hardware reliability as well as economics and social science (Rossi et al. 2010; Lyu 1996; Succi et al. 2003a). We let the reader refer to Appendix A for the full list of state-of-the-art models we selected. In particular, Table 10 lists them and provides the corresponding analytical expressions. As shown by the table, such expressions are parametric (either two or three parameters per individual model). Thus, non-linear regression allows the parameter values to be identified by fitting them on performance violations data, as described in the following.

For each operation *o*_*j*_, the observed performance violations (according to Equation 2) during a test session can be defined as follows:

$$X_{j}=\{(t_{n},n): RT_{j}(t_{n})>{\Gamma}_{j}\}$$

where (*t*_*n*_,*n*) is a pair composed of the timestamp of the *n*^*th*^ violation and *n*, the cumulative number of violations occurred up to *t*_*n*_. A candidate performance function *f* is differentiable and satisfies the following conditions:

1. $\frac {d f_{j}}{dt}>0$
2. $\exists t_{0} \text { s.t. } \forall t>t_{0}, \frac {d^{2}f_{j}}{dt^{2}}(t)<0$

**Fig. 6:**
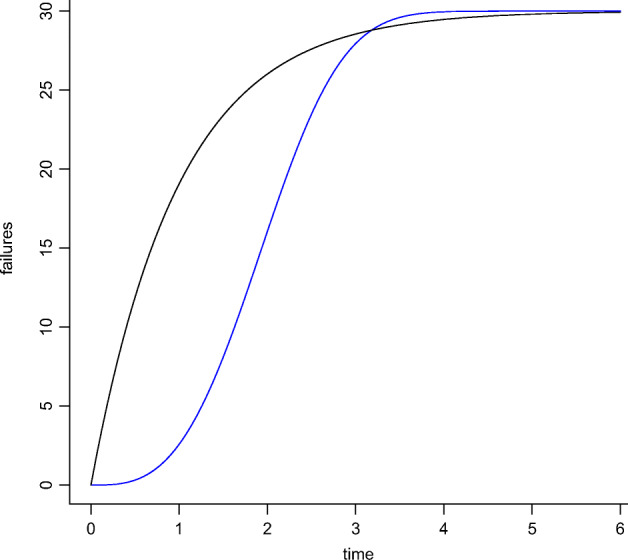
Example of concave and S-shaped models

Condition *i*) indicates that *f* grows monotonically, which means that the cumulative number of violations keeps growing over time. Condition *ii*) detects a change of concavity of the curve. The curve is convex before *t*_0_ and then concave after *t*_0_. This means that after *t*_0_ the occurrences of violations get sporadic as time goes by. In other words, the cumulative number of violations still increases but at a lower rate. Depending on whether *t*_0_ is the initial or any later time instant, the resulting curve is *concave* (i.e., the violation rate decreases from the beginning) or *S-shaped* (i.e., the violation rate increases and then decreases after a certain amount of time), as shown in Figure 6. An S-shaped curve has a flex at *t*_0_, i.e. the time instant at which the curve’s concavity changes. At this point, the curve experiences its maximal growth rate (highest violation rate). After this point, the curve becomes convex, thus the operation recovers and the violation rate starts decreasing (the time between two consecutive violations increases). After the flex, violations become rarer with time until they reduce to sporadic events. When *f*_*j*_ satisfies the two conditions, the operation *o*_*j*_ is considered *resilient* (i.e., it can recover from a performance issue). If *f*_*j*_ is also bounded (it has a horizontal asymptote *a*), the operation recovers from a performance issue in a finite amount of time, i.e., at time *t*_1_ in which the number of residual violations can be predicted by the model as *a* − *f*(*t*_1_). When the function *f* (e.g., a linear function) does not satisfy the condition *ii*), the related operation continuously experiences performance violations occurring with either constant or increasing rate. When the function *f* satisfies both conditions but it is not finite, we cannot tell whether the related operation is resilient in finite time as the number of residual violations cannot be calculated.

Since the analytic expressions in Table 10 represent *finite* models (Lyu 1996), an upper bound for the growth also exists (i.e., a horizontal asymptote). Intuitively, if the data gathered by testing the microservices yields a finite model (either concave or S-shaped), the degradation of performance is limited and can be overall estimated by an expected number of violations. As mentioned above, Figure 6 shows an example of two candidate growth models in the two cases: concave or S-shaped, respectively. The natural interpretation of a concave model in our context yields an immediate steep violations’ growth followed by its gradual decrease. The S-shape model exhibits instead a change in the pace of violations occurrences for which an operation has an initial low pace of violations’ occurrence and experience the steep growth only later.

### Calibration of performance models

**Fig. 7:**
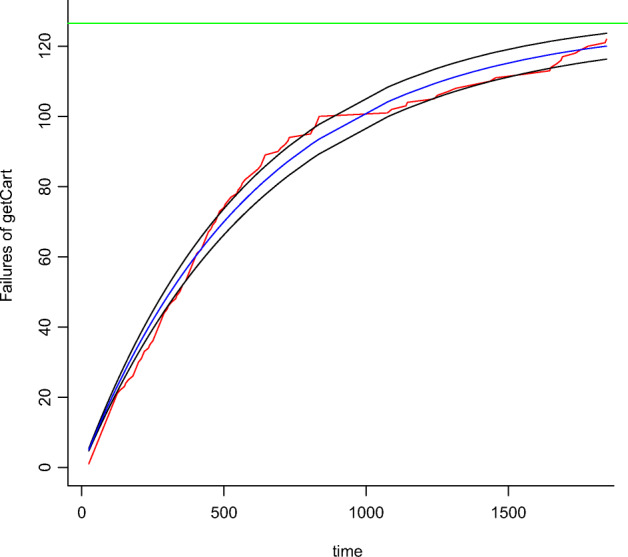
GO model for (blue line) calibrated from observations (red line)

We introduce here an approach we use to calibrate the growth models taking into account the data collected during operation. To determine the parameters’ values, state-of-the-art analytic models (see Table 10 in Appendix A) are fitted on performance violation data with *Ordinary Least Square* (OLS) method, as described by Lyu (1996) and Rossi et al. (2010). For instance, Figure 7 shows the Goel-Okumoto (GO) concave model (Goel and Okumoto 1979) fitted upon the performance violations of the microservice operation . The plot shows the observed violations (red line) and the calibrated model (blue line). The black lines surrounding the blue line delimit the area of the 95*%* Confidence Interval (CI). The fitting procedure starts by selecting initial values for all parameters (*a*_0_,*b*_0_,*c*_0_). This choice is nothing but trivial with non-linear regression and multiple parameters (Virene 1968; Huang et al. 2010). Indeed, a poor choice may result in a long iterative computation that may either diverge or converge towards a non-satisfactory local minimum. To mitigate these issues, we developed heuristics able to reduce the search space of initial values for the model’s parameters, as suggested by Virene (1968). To this aim, we analyzed each function in Table 10 in terms of its behavior as analytic expression as detailed below.

- Being the growth models finite, we estimated the initial value of the parameter *a* as the number of the total expected violations using the behavior of the model expression *f* at infinite and we set it proportional to the total number of observed violations *A* as follows:
  6 $$  \lim_{t \to \infty} f(t) = a \propto A  $$
  As an example, the total number of violations observed with is 120. We used this value to set our initial guess of the parameter *a* that was then calibrated to 127 (i.e., the green horizontal line in Figure 7).
- Given *t*_1_ and *t*_2_, two initial time values, we estimate the first derivative $f^{\prime }$ at 0 as the increment ratio of the function *f* between *t*_1_ and *t*_2_:
  7 $$  f^{\prime}(0)(b,c)\sim \frac{f(t_{2})-f(t_{1})}{t_{2}-t_{1}}  $$
  then, we compute the remaining parameters *b* and *c* (if exists^7^) by solving Equation 7. For instance, according to Figure 7, the number of violations at *t*_2_ = 5 is around 25. Thus, we can estimate the missing parameter *b* (the model GO has two parameters in total) by solving the inequality $f^{\prime }(0) \sim 0.2$.
- If needed, we then chose the values of the remaining parameter over a range of values according to the parameter constraints of the model as described in Table 10.

From such estimations, we restricted the search space of the initial parameters’ values and reduced the problem by iterating our procedure in Algorithm 1 over a finite sample of trial values (e.g., *x* ∈{1,...1000}). Algorithm 1 refines the regression process *K* times to search for significant parameters’ values in case the regression with the initial parameters’ values does not find a local minimum and parameters’ values are not significant (line 13). The calibration procedure implemented in R^8^ using the non-linear regression algorithm of the package nls2^9^. 
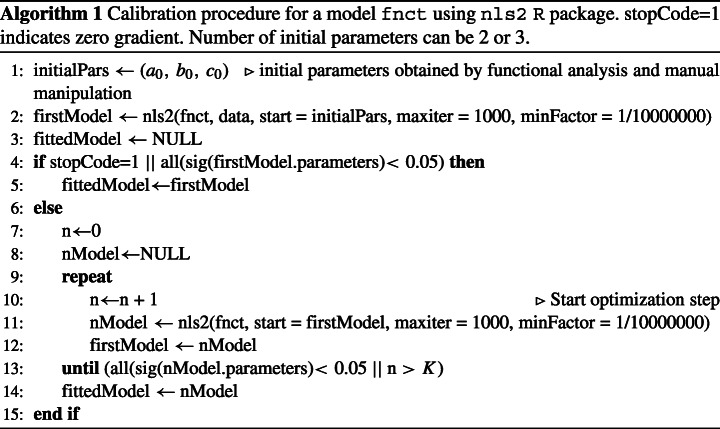


### Model selection

All the candidate models calibrated through the procedure reported above, are then ranked based on their ability to estimate and predict performance violations for each individual operation. Firstly, we evaluate how well observations are represented by the models. For this purpose, we use the *Coefficient of Determination* *R*^2^, which quantifies the proportion of total variation of observations explained by the model, see Table 11. As described by Glantz and Slinker (2001), this measure is known as the “goodness of fit” and the ideal (and maximum) value is *R*^2^ = 1. For non-linear models, *R*^2^ can also be negative. In this case, the mean of the data provides a better fit than the models. In addition, the measure does not take into consideration the number of model parameters as described by T.O. (1983), Spiess and Neumeyer (2010), and Rossi et al. (2010). For this reasons, we do not use such measure to compare the models but we use it to discard those models whose *R*^2^ value is lower than 0.95. Then, models are compared by means of four *accuracy measures*. Namely, two measures for *estimation ability* (Relative Precision of Fit (RPF) and Coverage of Fit (CoF)) and two measures for *prediction ability* (Predictive Ability (PA) and Accuracy of the Final Point (AFP)). Definitions and value ranges are reported in Appendix A, Table 11. The metrics RPF and CoF capture two complementary facets of the estimation ability of a fitted model: the size of the 95*%* CI and the number of observed violations captured in such area, respectively. Best models have minimum RPF (approaching the ideal value 0) and maximum CoF (approaching the ideal value 100*%*). The metrics AFP and PA quantify two complementary aspects of *prediction ability*. AFP yields the ability of the model to approach the total number of expected violations within the observation time. PA tells how early such approximation can occur. Considering the last two metrics, the top models are those associated with minimum PA (approaching the ideal value 0) and minimum AFP (approaching the ideal value 0).

We analyze each model first by using individual measures and then by combining RPF and CoF for estimation, and AFP and PA for prediction. In the latter case, we then rank models by means of the Euclidean distance from the ideal values, as follows:

8 $$  d_{E} = \sqrt{|\mathit{RPF}-0|^{2}+|\mathit{CoF}-1|^{2}}        d_{P} = \sqrt{|\mathit{PA}-0|^{2}+|\mathit{AFP}-0|^{2}}  $$

To avoid biases in using Euclidean distance for different scales, *d*_*E*_ and *d*_*P*_ have been computed after all measures and ideal values have been normalized in the range [0,1]. Normalization has been performed using measures’ min-max values on the bounded region surrounding the measures’ ideal values for which *AFP* ≤ 2, *CoF* ≥ 30*%* and *RPF* ≤ *d*_*o*_/3, with *d*_*o*_ total number of violations for the operation *o*^10^. Finally, we select the *top models* according to such rankings by considering either each individual measure or the Euclidean distance which allows engineers to easily spot models that exhibit a good trade-off between estimation and prediction.

## Experimental results

In this section, we discuss the major results obtained from our experiments to answer the research questions introduced in Section 3.

### RQ1: *What is the cost-effectiveness of the proposed calibration heuristic for fitting growth models?*

**Fig. 8:**
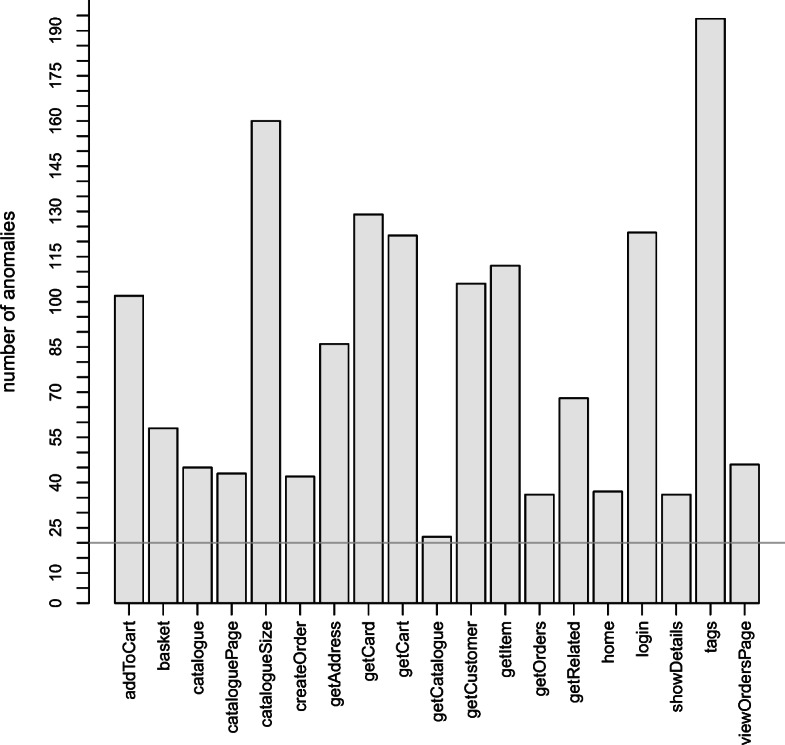
Performance violations of microservice operations collected during Test2

In the context of RQ1, we aim at studying the cost of the model calibration process as well as its effectiveness. This investigation is motivated by the fact that the application of our approach may be prohibitive in case such a cost is too high. To answer the question, we have collected the execution time and the success ratio of the calibration process conducted in our in-vitro experiments with Sock Shop. Figure 8 illustrates the bar plot of the number of observed violations per operation collected during Test2. The number significantly varies depending on how the operation is able to handle the requests. For instance, the number of violations observed for the operation is almost 200, whereas for it is almost 25. For all operations, we were able to collect at least 20 violations (horizontal line in Figure 8).

For each choice of the initial model parameters (*a*_0_,*b*_0_,*c*_0_), estimated as described in Section 4.3, and each combination of the 19 operations and nine growth models, we executed Algorithm 1 with *K* = 10 to fit the models. In total, we executed the calibration process 171 times. In 96*%* of the cases, we successfully obtained model convergence and parameters’ significance. In particular, for GOS and all operations, our heuristic has been able to directly identify parameter values starting from our initial estimation. For all remaining models but HD, W, and YR a multiplier of the initial estimation of one or more parameters was needed. For HD, W, and YR a number of operations (5, 8, and 3, respectively), we further needed educated guesses on the previous regression iterations to manually identify the initial values of the parameters. For only 4*%* of the cases, we did not achieve convergence within *K* = 10 iterations. Thus, we increased *K* by an order of magnitude (*K* = 100) to check whether convergence was feasible by increasing the overall effort. In all these cases we observed no convergence as outcome of the calibration. Table 2 shows the fitted parameters of each individual model and each operation. Empty cells correspond to not converging fitting process (4*%* of the cases), whereas grey cells indicate parameters’ values significant but outside the required ranges for the model (6*%* of the cases), as defined in Table 10 (e.g., negative values).

**Table 2:** Fitted parameters *a*, *b*, and *c* (when available) of the models reported in Table 10 per operation. Empty cells indicate no significant fitting, boldface indicates negative parameter values

service		GO	GOS	Gomp	HD	L	W	WS	YE	YR
	a	8.78e + 09	1.21e + 02	1.38e + 02	2.37e + 02	1.12e + 02	1.59e + 10	1.54e + 08	1.65e + 08	1.40e + 09
addToCart	b	6.49e-12	1.67e-03	4.66e-02	7.01e-04	1.21e + 01	2.05e-12	2.04e-05	**-2.61e-06**	6.98e-08
	c			9.99e-01	2.49e + 00	2.39e-03	1.07e + 00	5.37e-01	**-1.17e-04**	1.76e-06
	a	9.43e + 01	5.87e + 01	6.10e + 01	6.45e + 01	5.61e + 01	7.14e + 01	2.16e + 12	4.01e + 09	7.95e + 08
basket	b	5.32e-04	2.45e-03	5.66e-02	1.66e-03	9.44e + 00	2.51e-04	4.62e-06	2.35e-08	6.63e-08
	c			9.98e-01	1.63e + 00	3.25e-03	1.17e + 00	2.10e-02	5.32e-04	3.38e-06
	a	2.35e + 10	6.03e + 01	7.45e + 01		5.41e + 01		1.66e + 12	3.65e + 08	3.12e + 09
catalogue	b	9.94e-13	1.34e-03	3.93e-02		1.36e + 01		**-5.11e-06**	**-2.02e-07**	1.50e-08
	c			9.99e-01		2.14e-03		4.87e-03	**-2.54e-04**	1.26e-06
	a	2.95e + 10	8.68e + 01	7.19e + 01	6.07e + 01	5.09e + 01	1.26e + 02	1.72e + 12	1.77e + 08	6.67e + 08
cataloguePage	b	7.60e-13	9.31e-04	1.84e-02	1.83e-03	2.09e + 01	5.87e-06	**-4.89e-06**	**-1.85e-07**	8.09e-08
	c			9.99e-01	9.39e + 00	2.60e-03	1.49e + 00	4.82e-03	**-4.84e-04**	9.60e-07
	a	1.50e + 11	2.04e + 02	2.18e + 02	4.33e + 02	1.74e + 02	1.15e + 08	1.32e + 08	1.30e + 08	4.90e + 09
catalogueSize	b	5.57e-13	1.42e-03	3.71e-02	6.12e-04	1.27e + 01	4.01e-10	2.64e-05	**-4.25e-06**	3.21e-08
	c			9.99e-01	2.65e + 00	2.41e-03	1.08e + 00	5.41e-01	**-1.37e-04**	1.57e-06
	a	5.43e + 01	3.85e + 01	4.66e + 01	8.38e + 01	4.19e + 01	6.64e + 08	1.14e + 12	7.22e + 08	1.35e + 09
createOrder	b	7.00e-04	2.71e-03	1.00e-01	**-3.57e-10**	6.27e + 00	3.18e-10	**-4.67e-06**	7.53e-08	2.66e-08
	c			9.99e-01	**-1.00e + 0**	2.41e-03	7.00e-01	4.66e-02	7.01e-04	3.61e-06
	a	1.94e + 10	1.13e + 02	1.22e + 02		9.70e + 01	4.47e + 09	2.04e + 12	2.07e + 08	2.47e + 09
getAddress	b	2.24e-12	1.33e-03	3.50e-02		1.29e + 01	4.00e-12	5.84e-06	**-8.80e-07**	3.42e-08
	c			9.99e-01		2.24e-03	1.12e + 00	1.75e-02	**-2.05e-04**	1.46e-06
	a	6.85e + 02	1.37e + 02	1.68e + 02	1.29e + 03	1.40e + 02	2.24e + 08	1.17e + 13	3.44e + 08	5.95e + 08
getCard	b	1.09e-04	1.84e-03	5.68e-02	**-1.83e-10**	9.42e + 00	4.78e-10	1.66e-06	2.00e-06	1.95e-07
	c			9.99e-01	**-1.00e + 0**	2.22e-03	9.39e-01	1.10e-01	1.09e-04	2.16e-06
	a	1.27e + 02	1.12e + 02	1.13e + 02	1.15e + 02	1.10e + 02	5.51e + 08	1.55e + 13	8.21e + 09	2.44e + 09
getCart	b	1.61e-03	4.55e-03	5.31e-02	3.55e-03	9.35e + 00	3.58e-09	1.77e-06	1.54e-08	4.38e-08
	c			9.96e-01	1.88e + 00	5.85e-03	5.61e-01	8.29e-02	1.61e-03	1.04e-05
	a	9.70e + 01	2.42e + 01	3.13e + 01	1.80e + 02	2.46e + 01	4.08e + 08	8.15e + 09	6.11e + 08	2.34e + 08
getCatalogue	b	1.34e-04	1.77e-03	6.24e-02	**-2.22e-10**	8.98e + 00	5.50e-11	5.31e-05	1.58e-07	8.77e-08
	c			9.99e-01	**-1.00e + 0**	2.13e-03	9.11e-01	**-1.95e-05**	1.34e-04	1.95e-06
	a	1.26e + 10	1.34e + 02	1.38e + 02	1.55e + 02	1.14e + 02	1.08e + 08	1.01e + 08	9.39e + 08	1.33e + 09
getCustomer	b	4.54e-12	1.49e-03	3.60e-02	1.26e-03	1.29e + 01	3.36e-10	2.68e-05	4.75e-06	7.91e-08
	c			9.99e-01	3.32e + 00	2.53e-03	1.06e + 00	5.32e-01	1.29e-05	1.66e-06
	a	9.05e + 10	1.46e + 02	1.62e + 02	2.82e + 02	1.27e + 02	3.65e + 08	2.22e + 09	1.17e + 08	1.18e + 09
getItem	b	6.51e-13	1.40e-03	3.86e-02	7.20e-04	1.24e + 01	8.92e-11	2.16e-04	**-3.19e-06**	9.50e-08
	c			9.99e-01	3.36e + 00	2.29e-03	1.08e + 00	6.23e-03	**-1.42e-04**	1.42e-06
	a	3.85e + 01	3.18e + 01	3.66e + 01	5.44e + 01	3.41e + 01	1.34e + 08	5.67e + 09	1.18e + 09	1.41e + 09
getOrders	b	1.08e-03	3.34e-03	1.27e-01	**-3.35e-10**	5.17e + 00	3.38e-09	7.28e-05	3.27e-08	2.13e-08
	c			9.98e-01	**-1.00e + 0**	2.71e-03	5.77e-01	2.16e-02	1.08e-03	5.45e-06
	a	9.02e + 01	5.97e + 01	7.55e + 01	1.42e + 02	6.94e + 01	2.11e + 08	1.15e + 12	2.24e + 09	9.78e + 08
getRelated	b	6.12e-04	2.72e-03	9.81e-02	**-4.28e-10**	6.17e + 00	1.23e-09	6.86e-06	4.02e-08	5.59e-08
	c			9.99e-01	**-1.00e + 0**	2.08e-03	7.29e-01	2.09e-02	6.12e-04	4.29e-06
	a	8.20e + 09	8.81e + 01	1.01e + 02		5.74e + 01	1.79e + 09	1.18e + 09	**-6.19e + 8**	2.23e + 08
home	b	2.11e-12	7.33e-04	1.74e-02		2.02e + 01	4.63e-13	1.80e-04	2.70e-08	2.18e-07
	c			9.99e-01		1.88e-03	1.42e + 00	**-1.53e-04**	**-6.22e-4**	7.11e-07
	a	1.39e + 02	1.10e + 02	1.15e + 02	2.10e + 02	1.10e + 02		6.95e + 08		7.35e + 08
login	b	1.05e-03	3.60e-03	7.35e-02	**-1.45e-10**	7.23e + 00		5.13e-05		1.39e-07
	c			9.97e-01	**-1.00e + 0**	3.90e-03		3.29e-01		7.19e-06
	a	1.06e + 02	3.75e + 01	4.88e + 01	1.90e + 02	4.01e + 01	5.89e + 09	5.71e + 09	1.22e + 08	1.24e + 09
showDetails	b	2.14e-04	1.93e-03	6.72e-02	**-3.02e-10**	8.46e + 00	8.00e-12	8.02e-05	8.67e-07	2.62e-08
	c			9.99e-01	**-1.00e + 0**	2.10e-03	8.78e-01	5.08e-04	2.15e-04	2.20e-06
	a	3.86e + 11	2.51e + 02	2.85e + 02	5.37e + 02	2.20e + 02	1.24e + 08	6.97e + 12	1.48e + 09	4.59e + 08
tags	b	2.73e-13	1.46e-03	4.03e-02	5.98e-04	1.24e + 01	5.50e-10	4.94e-06	1.10e-05	4.25e-07
	c			9.99e-01	2.42e + 00	2.38e-03	1.06e + 00	1.14e-02	6.46e-06	1.60e-06
	a	6.61e + 09	5.24e + 01	7.30e + 01		5.71e + 01	3.27e + 08	5.95e + 11	2.95e + 08	3.72e + 08
viewOrdersPage	b	3.69e-12	1.65e-03	4.84e-02		1.06e + 01	6.51e-11	**-8.12e-06**	3.72e-06	1.14e-07
	c			9.99e-01		1.98e-03	1.02e + 00	1.49e-02	2.25e-05	1.91e-06

The execution of our calibration procedure for Sock Shop lasted in total 3 hours and 9 minutes. During this time, the algorithm was completely executed for 171 times (9x19). Thus, on average 1 hour and 6 minutes per microservice (Table 3).

**Table 3:** Fitting execution time within the in-vitro experiments using Sock Shop (time less than 1 second is ignored)

model	GO	GOS	Gomp	HD*	L	W*	WS	YE*	YR
total time	0:37:49	0:00:00	0:04:31	0:09:20	0:00:01	0:42:51	0:01:02	1:08:43	0:24:35
average time	0:01:59	0:00:00	0:00:14	0:00:29	0:00:00	0:02:15	0:00:03	0:03:37	0:01:18

^∗^ Non-converging fitting process for at least one operation.


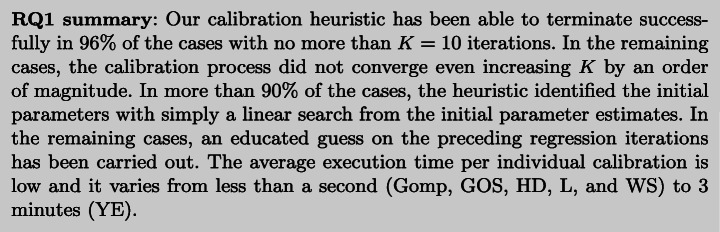


### **RQ2:***To what extent do the proposed non-linear growth models accurately estimate the occurrences of performance violations of microservice operations?*

To answer RQ2, we further analyzed the accuracy of the fitted non-linear models for the 19 services of Sock Shop and we compared it with the accuracy of the linear models.

**Fig. 9:**
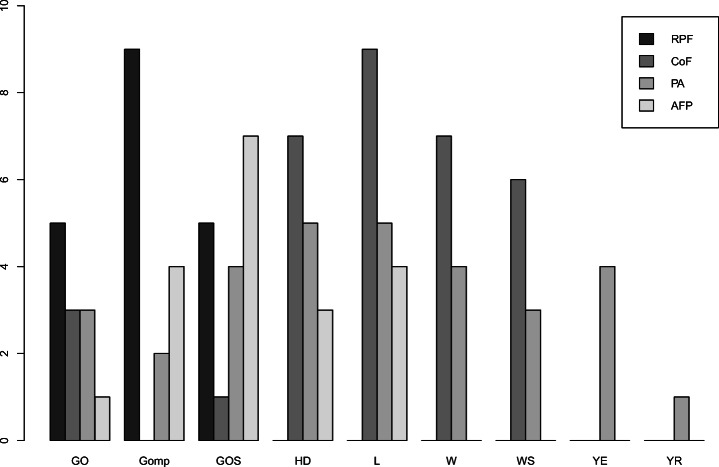
Number of top non-linear models per accuracy measure

To this aim, we first studied the top non-linear models for each accuracy measure, i.e., models that achieve the best value for the measure. Figure 9 shows that the microservice operations have different top non-linear models for RPF, CoF, PA, or AFP. For instance, GO, Gomp, and GOS, are top models for RPF and all operations, whereas considering CoF, top models for some operations are also HD and L. Then, we studied how the models trade-off among the different measures and we analyzed them in terms of their estimation and prediction ability defined by the two Euclidean distances *d*_*E*_ and *d*_*P*_, as described in Section 4.3.

Table 4 reports per operation the two models that have respectively the best *d*_*E*_ score (E-model) and *d*_*P*_ score (P-model). In this case, we can see that the logistic model L is the most accurate (i.e., best *d*_*E*_ and *d*_*P*_) for a larger set of operations.

**Table 4:** Top models per operation according to the rankings defined by *d*_*E*_ and *d*_*P*_ (E-model and P-model, respectively)

service	E-model	RPF	CoF	P-model	AFP	PA
addToCart	GO	2.93	52	GOS	0.15	0.93
basket	L	2.50	52	GOS	0.01	0.84
catalogue	L	6.72	74	L	0.18	0.96
cataloguePage	L	3.79	67	L	0.18	0.90
catalogueSize	L	8.78	68	L	0.09	0.92
createOrder	L	5.19	60	L	0.00	0.96
getAddress	GOS	9.38	79	L	0.13	0.95
getCard	GOS	11.51	60	L	0.09	0.94
getCart	GOS	3.65	53	HD	0.06	0.67
getCatalogue	L	4.51	77	L	0.12	0.93
getCustomer	L	5.53	57	L	0.08	0.89
getItem	GOS	10.19	76	GOS	0.28	0.95
getOrders	L	3.87	53	Gomp	0.02	0.90
getRelated	L	12.11	57	Gomp	0.11	0.00
home	NA	NA	NA	L	0.55	0.96
login	GO	6.98	58	L	0.12	0.00
showDetails	L	6.48	69	L	0.11	0.91
tags	L	8.14	64	L	0.13	0.88
viewOrdersPage	GOS	8.78	83	L	0.24	0.93

**Fig. 10:**
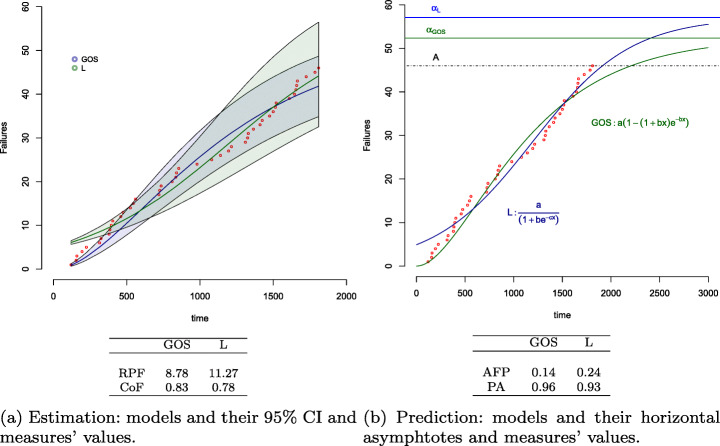
Visualization of GOS and L models for

To better understand the values of Table 4, we visualize the E-model GOS and P-Model L for , as an example, in Figure 10. Figure 10a plots violations (red dots) and the models GOS and L along with their 95*%* CI areas and their RPF and CoF values, whereas Figure 10b plots violations, the same models and their asymptote lines along with the line representing the total number of violations observed (*A*), and their AFP and PA values. GOS outperforms L in terms of estimation since it has a smaller CI area (the RPF is equal to 8.78 and 11.27, respectively) and it captures more violation data points at the beginning of the observation period (the CoF is equal to 0.83 and 0.78, respectively). The change in the shape of the two models is also different, as shown in Figure 10b. GOS starts decreasing the violation rate (curve flex) earlier at around 480 secs, whereas L does it at around 1000 secs. The total number predicted by the model GOS approximates better the total number of violations (PA= 0.14) whereas the model L predicts the total number of failures earlier (PA= 0.93), even though both models do not predict the total number of violations much in advance as PA in both cases is close to 1.0.

Finally, we fit the linear model on the data of each operation and compute the measures CoF, RPF, and PA^11^. The resulting values are reported in Table 5.

**Table 5:** Accuracy of the linear models

	*R*^2^	RPF	CoF	PA
addToCart	0.99	2.80	20	0.96
basket	1.00	2.82	36	0.86
catalogue	0.99	2.21	58.33	-
cataloguePage	0.93	10.99	32	-
catalogueSize	0.97	5.02	51	0.82
createOrder	0.98	4.68	50	-
getAddress	0.97	4.02	61	-
getCard	1.00	2.43	57	0.89
getCart	1.00	2.35	51	0.85
getCatalogue	0.99	1.76	77	-
getCustomer	0.83	17.67	25	-
getItem	1.00	3.20	32	0.95
getOrders	0.99	3.08	43	0.87
getRelated	0.98	3.23	74	-
home	1.00	2.41	30	0.87
login	0.98	4.01	74	0.72
showDetails	0.99	2.18	70	0.91
tags	0.97	5.28	55	0.96
viewOrdersPage	1.00	2.88	54	0.90

**Fig. 11:**
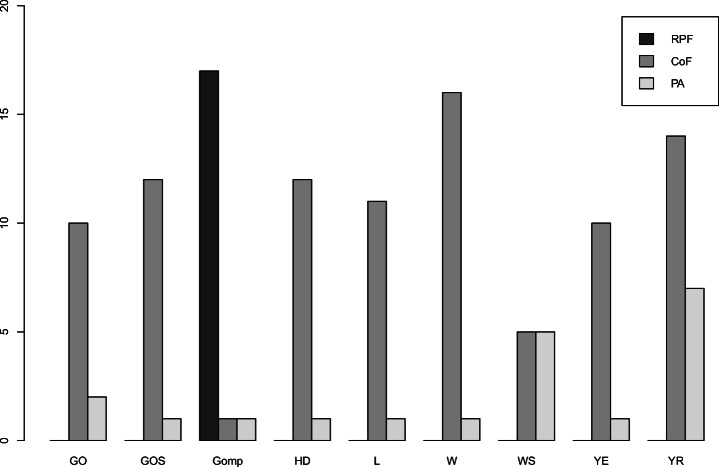
Number of operations for which non-linear models outperform the linear one in terms of RPF, CoF, and PA measures

By each individual measure, Gomp and W are better than the linear model in terms of RPF and CoF for all and almost all operations respectively, as shown in Figure 11. For PA, we found that 59*%* of the operations have a non-linear model that is more accurate than a linear one, but this model may vary across the operations.

**Fig. 12:**
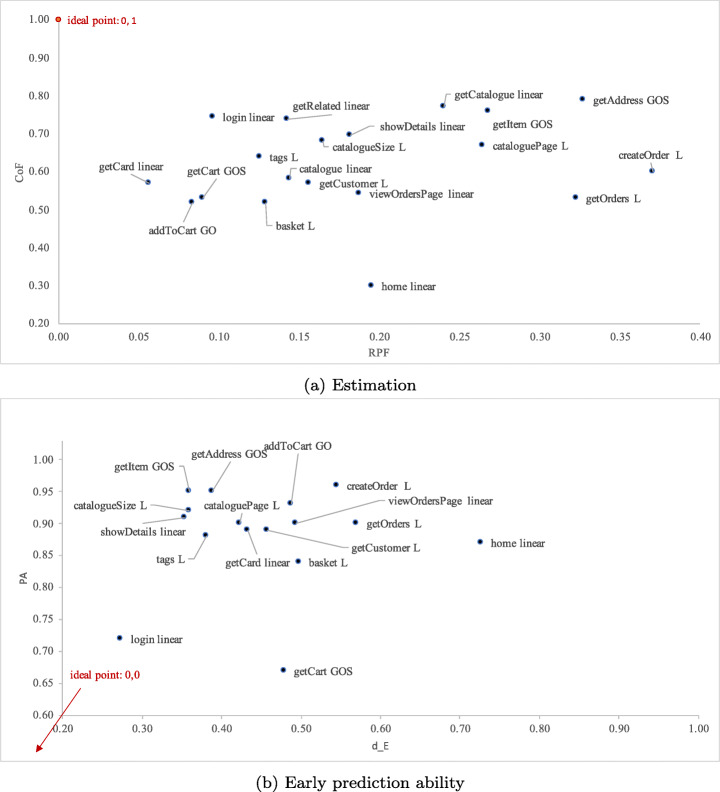
Linear or non-linear top model per microservice operation


As we are interested in trading off RPF and CoF, we then further compute *d*_*E*_ for the linear models (Table 5). We can see that there exists a small number of operations (3 out of 19) whose violations are better estimated by a linear model as shown in Figure 12a (i.e., ,, ). Linear models may also show a good ability for early prediction of the total number of observed violations, such as the operation that, as illustrated in Figure 12a and b, is closer to the ideal values of the measures compared to the other models.

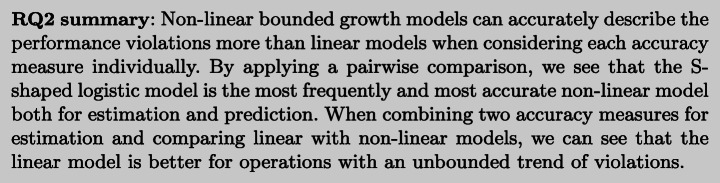


### **RQ3:***What are the insights we can derive from growth theory applied to the study of performance violations of a microservice system?*

We observed that *non-linear S-shaped finite models* represent the occurrences of violations for the majority of the operations of our SUT. As finite growth models are monotonic increasing and bounded, the time between two violations after a certain point increases with time and the total number of violations ever occurring for an operation is limited. An operation showing such behavior is better and better able to handle requests with due performance. Secondly, for the majority of the operations, the non-linear bounded models are also S-shaped (Table 5). In this case, the violations’ rate first increases until its maximal limit (at the flex) and then gradually decreases toward zero (at infinite). An operation showing such behavior is resilient: it can handle and recover from a degraded operating condition. For instance, when the gradual accumulation of the requests into the message queue eventually saturates causing a sudden increase of violations, the corresponding service may trigger adaptive countermeasures, like autoscaling that dynamically changes the number of microservice replicas and evenly distributes the load across them. In this case, the operation is able to recover its initial performance and the violations’ rate starts decreasing. Thus, the performance violations become more and more sporadic events.

We have also found that logistic is the growth model that represents the occurrences of performance violations in 7 out of the 19 microservice operations (Figure 12), but not for all of them. For some operations, the linear model is more accurate. In the following, we take a closer look at both the logistic and linear models.

As described by David and Edwards (2001), the mathematical expression of the logistic model^12^ can be used to represent a population with a bounded increase rate that depends on the population’s size. Rephrasing it in our context, such expression models the rate of occurrences of performance violations $\frac {d f}{d t}$ as proportional to the product of the cumulative number of occurred violations *f*(*t*) by time *t* and the violations not-yet occurred, *a* − *f*(*t*). The analytic expression of the rate is defined as follows:

9 $$  f^{\prime}(t)=\frac{d f(t)}{d t}=\frac{c}{a} \cdot f(t) \cdot (a-f(t))  $$

where *a* > 0 is the predicted total number of occurrences (i.e., the hypothetical upper-bound of the curve), and *c* > 0 is the increase rate. The analytic solution of the model L as in Table 4 is:

10 $$  f(t)=\frac{a}{(1+b \cdot e^{-c \cdot t})}  $$

with *b* > 1. According to our heuristic (Section 4.3), we estimated the initial parameters as in the following:

11 $$  a=A \cdot x,    b=\frac{a}{v}-1,    c= \frac{k}{v} \cdot \frac{a}{a-v}  $$

with $x \in \mathbb {R}_{>0}$, $k(t_{2},t_{1})=f^{\prime }(0)\sim \frac {f(t_{2})-f(t_{1})}{t_{2}-t_{1}}$ and *v* = *f*(0). The fitting process is then performed with the values reported in Table 6. by iterating Algorithm 1 over 1000 integer values for *x*. As described by Satoh and Yamada (2002), accurate estimates of the L parameters can be achieved if the dataset includes at least one point after the flex (i.e., the point in time at which the concavity of the model changes). According to the model L, the flex *t*^∗^ occurs when half of the total expected violations have occurred:

12 $$  t^{\ast} = \frac{log(b)}{c},   f(t^{\ast})=\frac{a}{2}  $$

**Table 6:** Values used for the fitting process of the model L

operation	x	*v*	(*t*_1_,*t*_2_)
addToCart, catologuePage	(1:1000)	1	(1,4)
getCart	(1:1000)	10	(1,2)
Remaining operations	(1:1000)	1	(1,2)

Thus, given the total number of observed violations *A*, we obtain a successful fitting process for the model L if the following condition holds:

13 $$ \begin{array}{@{}rcl@{}} \frac{a}{2} < A  \end{array} $$

For all our operations the inequality was satisfied^13^. For instance, the operation , for which L is the top model for both estimation and prediction, has *a* = 54.2 and *A* = 45 and Equation 13 is satisfied.

Equation 13 also provides a practical instrument for decision-making to guide online mechanisms of load balancing, like autoscaling. For instance, assuming that we have fitted offline the model, we can then monitor the number of violations when the system is in production, verifying whether this number is about to exceed half of the expected number of violations predicted by the model (i.e., *a*/2), and, thereafter, activate the autoscaling mechanism. Once the observed violations are fewer than half of the expected number of violations predicted by the model, the logistic model may not be distinguished from an unbounded model like the linear one.

The linear model represents even a preferable option for some of the operations of our system (e.g., ,, ). These operations are not able to improve the way they handle requests and violations keep on occurring at the same rate. Such operations yield performance issues and possible bottlenecks that need specific maintenance attention. 
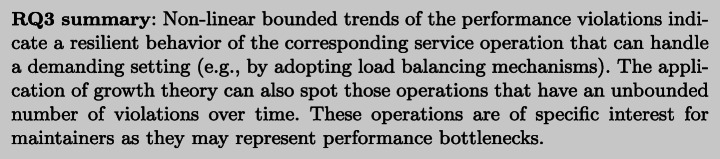


### **RQ4:***To what extent our methodology can be applied to real-world in-production systems?*

The goal of RQ4 is to illustrate how our methodology can also be applied to a real-world system running in production for which companies typically do not have much control over the operational context. As described in Section 3.3, we analyzed a Telecommunication system developed by Ericsson.

**Fig. 13:**
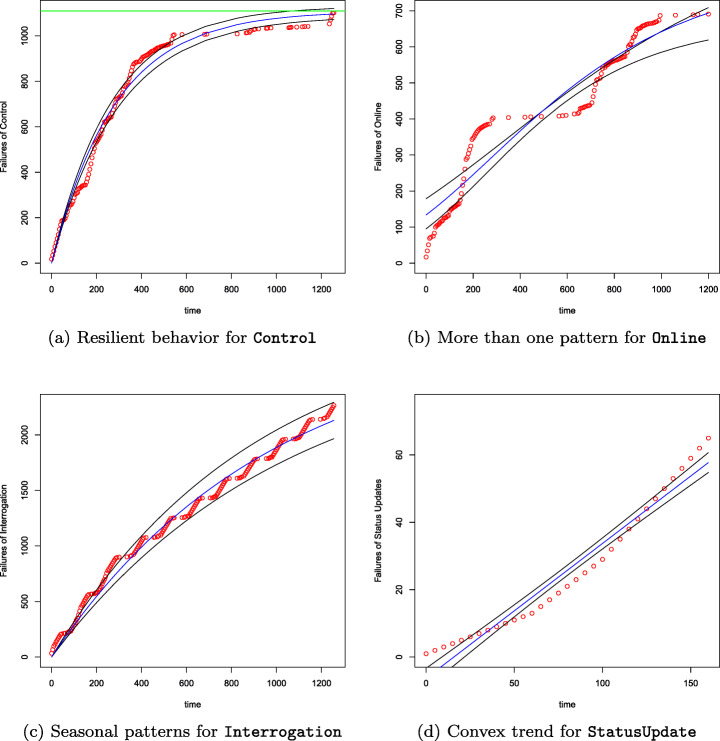
Types of trend of the performance violations observed by monitoring the microservices of the Telecommunication system. Each plot shows violations (red dots), a selected fitted model (blue line), and the corresponding CI (black lines)

Table 7 lists the number of violations per microservice collected during a session of $\sim 21$ hours. The table shows also the linear and non-linear fitted models. The execution of the calibration procedure for this system lasted in total 1 hour and 42 minutes. The calibration was completely executed 108 times (9 × 12). Thus, on average 57 minutes per microservice (9 minutes less than the execution time with Sock Shop). Table 7 shows that it was not always possible to fit all the linear and non-linear models with *R*^2^ ≥ 0.95 for all the microservices. This phenomenon can be explained by the fact that, during the period of observation, we found that the violations can follow additional trends than the basic concave/S-shaped one of the growth models observed for Sock Shop (see Figure 6). These additional trends are compositions of basic concave/S-shaped growth models. Figure 13 illustrates them: the basic concave/S-shaped growth model (Figure 13a), a combination of a few basic concave/S-shaped growth models (Figure 13b), a repeated periodical combination of the same basic concave/S-shaped growth model (Figure 13c), and a convex model (Figure 13d). Finally, Table 8 lists the top basic concave/S-Shaped models according to the *d*_*E*_ and *d*_*P*_ distances and their estimation and prediction measures. We can see that few models can estimate and predict the occurrences of violations. It is worth noting that for Interrogation, Recompose, and StatusUpdate the same model appears for estimation and prediction. These models have either high RPF or low CoF. By visualizing their graphs, we can see that the data does not follow a unique concave or S-shaped trend but rather a trend that joins multiple concave or S-shaped models. In other words, the application of our methodology to the Telecommunication system allows us to discuss different types of resilience behavior that were not found for Sock Shop, as follows.

**Table 7:** Fitted models with non negative parameters and *R*^2^ ≥ 0.95 per microservice

label	#violations	models
Adjustment	156	Linear and GOS, Gomp, L, W, YE
Control	1102	All non-linear models except WS
DBDataManagement	82	-
Enquiry	634	GO, GOS, Gomp
InternalCommunication	1125	All non-linear models except GOS, HD, WS, YR
Interrogation	2281	GO, Gomp
Offline	347	Linear
Online	685	Linear
Recompose	623	Gomp
ResourceRead	0	-
ResourceUpdate	0	-
StatusUpdate	66	Linear

**Table 8:** Top models and their break down on the estimation/prediction measures

microservice	E-model	RPF	CoF	P-model	AFP	PA
Adjustment	GOS	13.43	66.20	Gomp	0.23	0.89
Control	GOS	33.40	57.46	HD	0.05	0.46
DBDataManagement	-	-	-	-	-	-
Enquiry	GOS	20.50	54.76	GO	0.02	0.33
InternalCommunication	GO	122.01	77.51	Gomp	0.18	0.87
Interrogation	GO	227.12	78.86	GO	0.30	0.92
Offline	-	-	-	-	-	-
Online	-	-	-	-	-	-
Recompose	Gomp	9.12	22.30	Gomp	0.05	0.38
StatusUpdates	Linear	3.60	47.44	linear	-	0.98

#### Robust behavior

These microservices always meet the performance requirement. Thus, they can handle variations of server utilization up to their maximum. This is the case of the microservices and .

#### Resilient behavior with a single recovery profile

According to Table 7, 10 out of 12 services yield performance violations. In particular, the table lists those microservices for which the fitting and calibration process was successful. These services resemble the resilient behavior of most of the operations in Sock Shop. The total number of violations ever experienced by the microservice is limited and the rate of occurrences decreases in time after a certain instant and violations become more and more sporadic. An example is (Figure 13a), which exhibits a step increase of the violations and then a single recovery profile that eventually brings the microservice into acceptable behavior.

#### Resilient behavior with multiple recovery profiles

This is the case of the microservices , , and whose violations follow a trend that combines sequentially different basic growth models. For instance, Figure 13c illustrates the multiple recovery profiles for for which the model is a combination of four basic growth models with shapes (concave, concave, S-shaped, S-shaped). This is consistent with the findings reported by Avritzer et al. (2021) identifying these microservices as affected by extensive processing.

#### Resilient behavior with seasonal recovery profiless

This is the case of microservices and . For instance, the violations of occur with a seasonal basic concave model as shown in Figure 13c. According to Wert (2018), this represents the visible manifestation of the *application hiccup* antipattern which is typically caused by memory management issues (e.g., wrong cache usage, large temporary objects) which may lead to increased pollution of memory.

#### Non-resilient behavior

This is the case of for which the fitting process was successful only for the linear model. The collected violations follow a unbounded convex growth which indicates a growing rate of violation occurrences. 
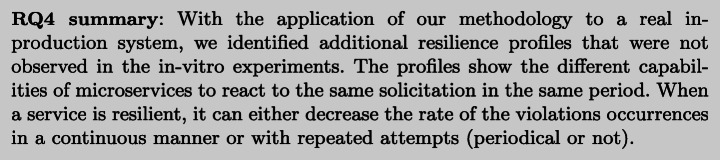


## Threats to validity

In this section, we discuss the major threats that could affect the validity of our work.

### Construct validity

The first threat is related to the strategy used to choose the threshold for performance violations. As introduced in Section 2, the choice of this threshold determines the number of violations occurring during the observation period. Thus, it might affect the significance of the regression analysis. We mitigated this threat by choosing a pass/fail criterion that ensured an adequate amount of violations leading to a significant regression analysis. We also detailed the criterion used in our experimental settings to allow replication of results.

A second threat is related to the fitting process. We used the Ordinary Least Square that fits the curve by finding the parameter values that minimize the difference between the data and the model function (i.e., the residuals). The difference is defined as the sum of the squared errors. This technique is generally considered the best one for small to medium sample sizes. Another viable approach is Maximum likelihood when the residuals have non-Gaussian distribution (Tamura and Yamada 2005). The maximum likelihood technique estimates parameters by solving a set of simultaneous equations that maximize the likelihood that the observed data came from a function with those parameter values. Maximum likelihood is generally considered a good statistical estimator in presence of a large sample size, that is not the case in our experimental setting. Furthermore, the CI provided by the maximum likelihood technique is usually more realistic because it is wider and asymmetric. Furthermore, the set of equations used to compute it is complex and must be solved numerically. As described by Wood (1996a), the least squares predictions are more stable and correlate slightly better with the original data.

### Internal validity

Even though such threats typically do not affect exploratory studies like the one presented in this article, we provide the reader with a detailed explanation of the experimental settings (both in-vitro and in-production) to increase the trustworthiness of our findings in terms of cause-effect relations. It is worth noting that our in-vitro experimental setting allowed for direct manipulation of the important factors of interest for our study: workload intensity, usage profile, deployment configuration, and the parametric pass/fail criterion which defines the performance requirements. The in-production experimental setting conducted with the case study at Ericsson allowed us to further study the applicability of our approach and generalize the findings to real-world systems. In both cases (in-vitro and in-production settings), we designed the experimental campaign by detailing all these factors to avoid possible misinterpretation of cause-effect relations.

### Conclusion validity

One of the typical threats of using the growth theory concerns future predictions. If the in-vitro testing environment in which the performance of the SUT has been observed changes considerably from the one used for the production, we cannot expect that the fitted models predict future performance in the same manner as stated by Pedrycz et al. (2012). This threat is partially mitigated by assuming that our experimental setting replicates the nominal conditions observed in production with high fidelity. Within the in-production experiments carried out at Ericsson, we replicated the nominal setting declared by the stakeholders of the company to extract the performance requirements. Then, we monitored the system while interacting with real end-users.

### External validity

Threats to external validity have been addressed by selecting a common case study in our target application domain, as described in Section 3.2.1. As stated by Avritzer et al. (2020b), Sock Shop has been recognized as a representative benchmark by the performance engineering community. Furthermore, we applied our approach to a real world system running in its production environment to study the applicability and generalize of our findings. Further generalization in other application domains requires additional experimental activities with a diverse set of case studies.

## Related work

The work presented in this paper has been influenced by existing work in the following major research areas: *performance modeling* of microservices systems, *growth theory* for event occurrences, and measures of *accuracy* and *prediction*.

### Performance modeling of microservices systems

A comprehensive literature review of microservice architectural challenges has been introduced in Alshuqayran et al. (2016). The authors focus on the specific challenges of this architectural style and related quality attributes. Such attributes for microservice architectures are mainly concerned with scalability, reusability, performance, fast agile development, and maintainability. A systematic gray literature review of the existing technical/operational pains and gains associated with microservices can be found in Soldani et al. (2018). According to this latter work, microservices research is still immature and there is a need for additional experimental and empirical evaluation of systematic methods and techniques able to support the life cycle of microservices systems. The survey also recognizes performance assessment of microservices as a pain in the IT industry.

Even though performance modeling has gained considerable attention by the software and systems engineering community in the past two decades, recent research activities highlight that performance delivered by microservices systems is hard to predict (Mukherjee et al. 2014; Soldani et al. 2018). This statement points to the necessity to capture performance evolution systematically and accurately. The detection of bottleneck components represents a common yet essential task for increasing the performance of microservices systems. A comprehensive survey on successful performance modeling approaches particularly tailored to component-based software systems can be found in Koziolek (2010). Performance modeling and prediction approaches have been proposed at the beginning for early design-time validation activities through simulations. The recent uptrend sees modeling interleaved with runtime stages, where specifications are constantly learned or incrementally built by observing runtime evidence as described by Calinescu et al. (2011). According to Avritzer et al. (2021), statistical characterization of software performance antipatterns can be extracted from the operational data by tuning *single server queuing models* (Bertsekas and Gallager 1992). This latter work focuses on those antipatterns that can be detected by analyzing steady-state behaviors of the target system, rather than looking at the performance evolution over time. Overall, automated approaches able to extract performance models from operational data are highly demanded, since they can estimate and predict the performance of a target implementation. As stated by Heinrich et al. (2017), among the key challenges that emerged in performance modeling for microservices are: finding appropriate modeling abstractions, and automated extraction of performance models. Our modeling methodology aims at tackling these major challenges and providing engineers with suitable techniques and tools able to aid performance engineering activities for microservices systems.

The work presented by Avritzer et al. (2020b) and Avritzer et al. (2018) introduced the concept of microservice failure in terms of violation of performance requirements. Violations are then aggregated to carry out the so-called Domain Metric based analysis that aims at evaluating the performance of the target microservices system as a whole. Specifically, the authors designed test cases by setting a testing pass/fail criterion through a performance threshold. The threshold is computed for each microservice by observing the SUT under a simulated usage profile, low load (two concurrent users), and high resources. The authors executed a series of such tests and measured the total performance of a SUT by computing the average response time of non-failing microservices. As described by Camilli et al. (2020), the Domain Metric approach has been also successfully adopted to evaluate a migration from monolithic systems to microservices. This latter work introduces an iterative methodology to recognize whether a migration step represents an improvement in terms of performance and scalability by performing a quantitative evaluation of alternative architectures. In our proposal, we define the pass/fail criterion to collect performance violations as in Avritzer et al. (2018) and Camilli et al. (2020). We build upon the notions of performance requirement as well as violation to analyze them over time and eventually build suitable models able to describe present occurrences and (when possible) predict future occurrences. To achieve this goal, performance violations for each microservice are modeled through a stochastic point process grounded on growth theory.

### Growth theory

A wide variety of growth models have been proposed to capture different facets of the fault detection process in software systems. Such models are traditionally used to describe the evolution of software reliability over time as reported by Port and Taber (2018), Taber and Port (2014), Rossi et al. (2010), Li et al. (2004), Li et al. (2005), Succi et al. (2003a), Stringfellow and Andrews (2002), and Biffl and Gutjahr (2002) and help decision-makers during software maintenance as in Taber and Port (2016). In this context, reliability is modeled with time of failure occurrences, which in turn are assumed to grow in time in such a way that the time to the next failure increases with time as well Musa et al. (1987), Bassin and Santhanam (1997), Lyu (1996), Succi et al. (2003a), Rossi et al. (2010), and Kumar et al. (2019). This behavior is modeled with stochastic processes that describe the cumulative number of failures over time (Rigdon and Basu 2000). The expected mean of the stochastic process defines a parametric function of time and represents the expected total number of failures at any time instant. Then the parameters of the expected mean are determined by non-linear regression on the actual data set of the cumulative number of failures over time.

Growth models based on non-homogeneous Poisson processes (Rigdon and Basu 2000) are particularly popular, such as the one proposed by Goel and Okumoto (1979). This model was introduced to describe the fault detection process as an exponential distribution. Other common proposals are the *Weibull* distribution (Goel 1985) and the S-shaped model introduced by Yamada et al. (1983). These models have been proposed to capture possible increasing or decreasing failure rates. Such a pattern is also followed by the log-logistic model introduced by Gokhale and Trivedi (1998), which describes an initial slow learning phase of the process. The logistic model can be used to model both concave and S-shape trends of the data. These days, such a model has shown its potential also in the context of the COVID-19 pandemic to model the contagion growth, as described by Shen (2020). The Gompertz model proposed by Ohishi et al. (2005) is more recent and it has been derived from the extreme value theory. Among the aforementioned models used for software reliability growth, the *Weibull* model turned out to be a good description of small software projects, as described by Li et al. (2004), Li et al. (2005), Rahmani et al. (2009), and Zhou and Davis (2005), whereas more complex models may be better to model large distributed systems (Tamura and Yamada 2005). The work introduced by Rossi et al. (2010) extends the previous study of Li et al. (20031) and Succi et al. (2003a) by combining the two approaches: software reliability growth across software versions and measures of accuracy and prediction. Again, the Weibull model outperforms other models across versions in terms of fitting and outliers.

Growth theory has been also used more recently to derive an empirical characterization of the debugging process as described by Cinque et al. (2017). As an example, the work presented by Nguyen et al. (2012) leverages growth theory to show that the time to repair is influenced by developer expertise and by the specific application context. The study carried out by Zhang et al. (2012) focused instead on the factors influencing the time latency between the bug assignment and the actual starting of the repair actions. Essentially, they found that the major factors are the assigned severity, the bug description, the number of methods, and the number of code changes.

To the best of our knowledge, the usage of growth theory to assess and predict performance violations of microservices systems has not been investigated yet.

### Accuracy of growth models

The measures of accuracy capture the properties of the growth models based on their ability to fit the collected data as well as the ability to predict forthcoming data. As described by Rossi et al. (2010), these measures can be categorized into three main groups: goodness of fit; precision of fit; and prediction ability. Both goodness and precision of fit refer to how the collected data is modeled, whereas the third category characterizes the ability of the model to predict on new future data. Common measures for the goodness of fit are the Coefficient of Determination, *R*^2^, (Draper and Smith (1998)) and the Akaike Information Criterion, Li et al. (2005). The Relative Precision of Fit and the Coverage of Fit represent two measures of choice for the precision of fit. They essentially provide the extent and the ability to capture the data in the 95*%* confidence interval of the model, as described by Succi et al. (2003b), and Wood (1996b), respectively. Measures of forecasting ability define the ability to predict early through the Predictive ability as described by Succi et al. (2003b), or accurately through the Accuracy of the Final Point, as in Yamada et al. (1983). These measures are typically used in combination as they give complementary information (see Section 4.3).

In this work, we also follow the guidelines introduced by Iannino et al. (1984) to carry out the evaluation of the candidate growth models. In particular, the following major criteria have been used: predictive ability (i.e., the ability of a model to predict future performance violations), capability (i.e., the capability of the model to estimate the occurring performance violations), applicability (i.e., a model should be applicable across different microservices’ operations of the SUT), and simplicity (i.e., in gathering data for its fitting, and its interpretation).

### Comparison with existing approaches

Here we present a comparison between our approach and selected state-of-the-art approaches that were recently introduced and that can be used to detect and study performance violations in the context of microservices or more in general service-based systems. Table 9 presents these approaches categorized into: main scope, theoretical foundation, granularity of detected violations, performance threshold, and SUTs that have been used as a benchmark for the corresponding approach. To the best of our knowledge, our approach is the first one that leverages the theoretical foundation of the growth models to study performance violations over time.

**Table 9:** Qualitative comparison among recent approaches able to detect performance violations

work	scope	approach	granularity	performance threshold	SUT
our approach	Study performance violations over time under unexpected workload conditions.	Chebyshev inequality, Growth theory	service level, individual requests	response time >T, T baseline requirement	Sock shop, Telecom system
Avritzer et al. (2018)	Study the scalability of different deployment configurations.	Chebyshev inequality, Domain-based metric	service level, aggregated requests	mean(response time) >T, T baseline requirement	Sock Shop
Avritzer et al. (2020b)	Study the scalability of different deployment configurations and the performance of the system under DoS attacks.	Chebyshev inequality, Domain-based metric	service level, aggregated requests	mean(response time) >T, T baseline requirement	Sock Shop
Camilli et al. (2020)	Study the performance of alternative architectures during the migration from a monolith to microservices.	Chebyshev inequality, Domain-based metric	service level, aggregated requests	mean(response time) >T, T baseline requirement	Smart mobility
Wert (2018)	Study the response time to detect performance issues and possibly map the issues to software performance antipatterns.	Parametric heuristics	service level, aggregated requests	P(response time >T) >p T, p given thresholds	Online Banking
Avritzer et al. (2021)	Study the response time to detect performance issues and possibly map the issues to software performance antipatterns.	Queuing systems, heuristics	service level, aggregated requests	max response time >T, T baseline requirement	Telecom system

The approaches introduced by Avritzer et al. (2018), Avritzer et al. (2020a), and Camilli et al. (2020) do not detect punctual violations but they aim at recognizing whether the average response time represents an outlier value. As reported in Table 9, the granularity of these latter is coarse-grained. Issues are still detected at the service level, but responses are collected and then aggregated, rather than being analyzed individually. The approach proposed by Wert (2018) detects individual performance violations with respect to given thresholds (e.g., service-level agreement). This setting could be applied in principle also in our approach. Nevertheless, as stated by Jiang and Hassan (2015), threshold values for non-functional requirements are often informally defined, leading testers to use rules of thumb (like the “no-worse-than-before” principle). In our case, formal performance requirements were not defined for both the Sock Shop benchmark and the telecommunication systems. For this reason, we systematically extracted the baseline requirements from the available data in a mechanical way by following the approach in Section 4. The analysis of introduced by Wert (2018) differs from our approach since it aims at constructing the empirical cumulative distribution of the response time per service to understand whether the probability of observing a response time greater than the given baseline threshold is acceptable. In addition, the parametric algorithms proposed by Wert do not provide a method to evaluate the parameters, quantify the performance requirements, and identify their violations.

The growth models used in this work (see Table 10) are models traditionally used in the context of reliability growth. Most of them (all models but L and Gomp) are non-homogeneous Poisson processes that gained popularity for describing the stochastic behavior of software faults detected during the testing phase. As described by Shibata et al. (2007), these models focus only on the fault-detection profile. In case, correction data is available, queuing models have been adopted to describe the fault-correction process in addition to detection. A popular model in this context is $M_{t}/M/\infty $ queue which assumes a time-dependent Markovian arrival, an exponential service, and an infinite number of fault correction personnel. It is worth noting that in the context of performance violations we cannot map the notion of fault-correction. Indeed, violations just occur. Thus, we can consider them as “instantly served”. This corresponds to a $M_{t}/D/\infty $ queuing model with constant service time and infinite resources. According to Shibata et al. (2007), this model coincides with a non-homogeneous Poisson growth model, which implicitly assumes an instantaneous fault-correction activity. According to our experience with in-vitro experiments reported in Section 5, we found that the performance violations of services exposed by Sock Shop are in $\sim 84\%$ of the cases better explained by the S-shaped non-linear model L (both in terms of estimation and prediction), rather than non-homogeneous Poisson processes falling in the category $M_{t}/D/\infty $.

Consistently with the findings reported for the in-vitro experiments, with the real-world in-production system, we observed that the violations associated with microservices having a resilient behavior are better described by S-shaped non-linear models. Non-resilient operations were identified in all the cases a simple linear regression was better than non-linear growth models. By analyzing the time series of the performance violations we also found seasonal patterns revealing the presence of application hiccups. It is worth noting that these latter issues were not detected using the approach presented by Avritzer et al. (2021) since, as shown in Table 9, the level of the approach is coarse-grained. Namely, it adopts aggregated performance indices (considering max response time values), whereas the application hiccup requires the analysis of the whole trend of the performance indices over time.

## Conclusions and future work

In this paper, we presented a novel approach to model transient performance behavior of microservice operations. Specifically, we have studied the time series of performance violations of microservice operations using growth theory. The main stages of the approach are as follows: *i*) *experiment design and execution*, which guides engineers in the definition of appropriate controlled experiments conducted adopting either in-vitro or in-production settings; and *ii*) *model fitting and selection*, whose major aim is to build and identify the best growth model(s) able to describe and possibly predict the performance evolution of each microservice (operation). We applied our approach using an in-vitro setting by testing a benchmark e-commerce system. We then replicated the experiments adopting an in-production setting by monitoring a real-world telecommunication system developed by Ericsson and running in the production environment of the company.

Our major results suggest that growth theory provides the foundation to model transient performance degradation of microservices and it provides engineers with practical insights derived by interpreting the analytic expression of the models. In our experience, non-linear bounded S-shaped growth functions describe the occurrence of performance violations better than linear models when the microservice can handle changes of the nominal operational setting and therefore eventually restore the ability to exhibit acceptable performance levels (e.g., by activating load balancing or horizontal scaling mechanisms). Our approach was also able to spot microservices whose performance behavior is not resilient. In this case, they exhibit a constant (or even exponential) growth of performance violations. For this reason, these microservices represent bottlenecks that need attention by engineers during system maintenance. The application of our methodology to a real in-production system identified additional resilience profiles that were not observed in the in-vitro experiments. These profiles show the ability of microservices to react differently to the same solicitation in the same time interval. We found that when a service is resilient, it can either decrease the violation rate in a continuous manner or with repeated attempts (periodical or not).

We plan to further explore how the fitted models can be used to predict future performance behavior of the microservice operations. We are going to investigate the applicability of traditional approaches used in software reliability analysis. For instance, exponential smoothing on datasets of multiple testing sessions (Li et al. 2004) has the potential of extrapolating the initial values of the model parameters with high accuracy. In this direction, other approaches used in reliability engineering, such as metaheuristic optimizing search might be explored as well (Antoniol et al. 2008; Benaddy et al. 2011).

## Appendix: A: Finite Growth Models and Metrics

Table 10 contains the definition of 9 state-of-the-art non-linear finite growth models we considered in this work. For each model, the table shows the equation of the curve and a textual description of the model parameters. We refer the reader to the provided references for a comprehensive discussion of these models. Table 11 illustrates the metrics used to rank and compare fitted models considering their estimation and prediction ability.

**Table 10:** Finite Growth Models

Model and type	Equation	Description
Goel-Okumoto (GO); Goel and Okumoto (1979); Concave	*a*(1 − *e*^−*b**t*^)	*a*: expected cumulative number of failures;
	*a* ≥ 0,*b* > 0	*b*: failure rate.
GO S-shaped (GOS); Yamada et al. (1983); S-shaped	*a*(1 − (1 + *b**t*)*e*^−*b**t*^)	*a*: expected cumulative number of failures;
	*a* ≥ 0,*b* > 0	*b*: failure rate.
Gompertz (Gomp); Virene (1968); S-shaped	${ab}^{{c}^{{t}}}$	*a*: expected cumulative number of failures;
	0 < *a* ≤ 1, 0 < *b* < 1, 0 < *c* < 1	*a**b*: initial reliability;
		*c*: the growth pattern indicator (small values of *c* indicate rapid early growth and large values of *c* indicate slow growth).
Hossain and Dahiya (1993); S-shaped	*a*(1 − *e*^−*b**t*^)/(1 + *c**e*^−*b**t*^)	*a*: expected cumulative number of failures;
	*a* ≥ 0,*b* > 0,*c* > 0	*b*: failure rate; *c*: ratio of detectable failures over the total final number of failures.
Logistic (L); Kececioglu (1991); S-shaped/Concave	*a*/(1 + *b**e*^−*c**t*^)	*a*: expected cumulative number of failures;
	*a* > 0,*b* > 1,*c* > 0	*b*: failure rate; for *b* > 1 S-shape otherwise concave; *c*: ratio of detectable failures over the total final number of failures.
Weibull (W); Goel (1985); S-shaped	$a(1-e^{-bt^{c}}),$	*a*: expected cumulative number of failures;
	*a* ≥ 0,*b* > 0,*c* > 0	*b*: failure rate; *c*: variation of failure rate.
Weibull more S-shaped (WS); Iannino and Musa (1990); S-shaped	$a(1-(1+bt^{c})e^{-bt^{c}})$	*a*: expected cumulative number of failures;
	*a* ≥ 0,*b* > 0,*c* > 0	*b*: failure rate; *c*: variation of failure rate.
Yamada Exponential (YE); Yamada et al. (1986); Concave	$a(1-e^{-b(1-e^{-ct})})$	*a*: expected cumulative number of failures;
	*a* ≥ 0,*b* > 0,*c* > 0	(*b*(1 − *e*^−*c**t*^)): cumulative testing effort based on exponential model.
Yamada Raleigh (YR); Yamada et al. (1986); S-shaped	$a(1-e^{-b(1-e^{-ct^{2}/2})})$	*a*: expected cumulative number of failures;
	*a* ≥ 0,*b* > 0,*c* > 0	$b(1-e^{-ct^{2}/2})$: cumulative testing effort based on Weibull model.

**Table 11:** Measures of Accuracy (Rossi et al. 2010)

Measure	Formula	Description and value range
Goodness of fit (*R*^2^)	$1-\frac {{\sum }_{i}(y_{i}-f(t_{i}))^{2}}{{\sum }_{i}(y_{i}-\bar {y})^{2}}$	Coefficient of Determination. How good is the model to approach the observed data w.r.t. the mean of the observed data $\bar {y}$; maximum ≤ 1. As suggested in Spiess and Neumeyer (2010), we use such measure only to select models with *R*^2^ < 0.95
Relative precision of fit (RPF)	$\frac {Area(CI)}{|T|}$	Size of the 95% Confidence Interval (CI) of the fitted curve normalized with the size of the interval of time service delivered; complementary to CoF; minimum ≥ 0
Coverage of fit (CoF)	$100 \cdot \frac {|\{ y_{i} \in Area(CI)\}|}{A}$	Percentage of data captured by the 95% CI; complementary to RPF; maximum in (0%,100%)
Predictive ability (PA)	$\frac {\min \limits _{t \in T}{ \{|A - f(t)|< 10\% A\}}}{T}$	Portion of time needed by the model to approach the total final number of observed failures, A; complementary to AFP; minimum in (0,1)
Accuracy of final point (AFP)	$\frac {|A-\alpha |}{A}$	Portion of remaining failures (defect slippage). A and *α* are respectively the observed and the predicted final total number of failures; complementary to PA; minimum ≥ 0

## Footnotes

1 Sock Shop is an open source project maintained by Weaveworks. Both the documentation and the sources are publicly available at https://microservices-demo.github.io/.

2 The software implementation of PPTAM as well as the dataset of our experiments are publicly available at https://github.com/pptam/pptam-tool

3 https://www.vmware.com/products/esxi-and-esx.html

4 http://www.emc-storage.co.uk/emc-vnx-5400

5 The complete specification of the deployment architecture of the production environment is omitted due to the disclosure agreement signed with Ericsson.

6 The names of the services have been abstracted by the Ericsson stakeholders according to the disclosure agreement.

7 The parameters of the models in in Table 10 are either two or three.

8 https://www.r-project.org/

9 https://cran.r-project.org/web/packages/nls2/

10 The region has been defined empirically to restrict the search by discarding poor models.

11 AFP cannot be computed for unbounded models.

12 Nowadays, L is making the headlines for being able to model the pandemic contagion of COVID-19, as illustrated in Shen (2020).

13 see the sample sizes in Figure 8 and the estimation of parameter a for the logistic model in Table 2.
